# scNCL: transferring labels from scRNA-seq to scATAC-seq data with neighborhood contrastive regularization

**DOI:** 10.1093/bioinformatics/btad505

**Published:** 2023-08-16

**Authors:** Xuhua Yan, Ruiqing Zheng, Jinmiao Chen, Min Li

**Affiliations:** School of Computer Science and Engineering, Central South University, Changsha 410083, China; School of Computer Science and Engineering, Central South University, Changsha 410083, China; Singapore Immunology Network (SIgN), Agency for Science, Technology and Research (A*STAR), Singapore 138648, Singapore; Immunology Translational Research Program, Department of Microbiology and Immunology, Yong Loo Lin School of Medicine, National University of Singapore (NUS), Singapore 117545, Singapore; School of Computer Science and Engineering, Central South University, Changsha 410083, China

## Abstract

**Motivation:**

scATAC-seq has enabled chromatin accessibility landscape profiling at the single-cell level, providing opportunities for determining cell-type-specific regulation codes. However, high dimension, extreme sparsity, and large scale of scATAC-seq data have posed great challenges to cell-type identification. Thus, there has been a growing interest in leveraging the well-annotated scRNA-seq data to help annotate scATAC-seq data. However, substantial computational obstacles remain to transfer information from scRNA-seq to scATAC-seq, especially for their heterogeneous features.

**Results:**

We propose a new transfer learning method, scNCL, which utilizes prior knowledge and contrastive learning to tackle the problem of heterogeneous features. Briefly, scNCL transforms scATAC-seq features into gene activity matrix based on prior knowledge. Since feature transformation can cause information loss, scNCL introduces neighborhood contrastive learning to preserve the neighborhood structure of scATAC-seq cells in raw feature space. To learn transferable latent features, scNCL uses a feature projection loss and an alignment loss to harmonize embeddings between scRNA-seq and scATAC-seq. Experiments on various datasets demonstrated that scNCL not only realizes accurate and robust label transfer for common types, but also achieves reliable detection of novel types. scNCL is also computationally efficient and scalable to million-scale datasets. Moreover, we prove scNCL can help refine cell-type annotations in existing scATAC-seq atlases.

**Availability and implementation:**

The source code and data used in this paper can be found in https://github.com/CSUBioGroup/scNCL-release.

## 1 Introduction

Recent advances in single-cell high-throughput sequencing technologies have enabled the emergence of diverse experimental methods that are capable of characterizing different properties of single cells. Single-cell RNA-sequencing (scRNA-seq) is the most widely used technique for the characterization of complex tissues and organisms at the single-cell level (Treutlein *et al.* 2014, Qiu *et al.* 2017, Rozenblatt-Rosen *et al.* 2017, Zheng *et al.* 2019). In addition, several technologies (Zong *et al.* 2012, Grosselin *et al.* 2019) have been developed to profile molecules other than the transcriptome in individual cells, such as chromatin accessibility and methylation. In particular, single-cell ATAC-seq (scATAC-seq) is an epigenomic profiling technique for measuring chromatin accessibility, which delivers a complementary layer of information to scRNA-seq and helps to understand epigenetic heterogeneity in complex tissues (Yu *et al.* 2020, Stuart *et al.* 2021). However, inherent sparsity, high dimension, and increasing size of scATAC-seq data have posed significant challenges in cell-type identification (Chen *et al.* 2019).

Fortunately, large amounts of scRNA-seq datasets have been well-annotated (Rozenblatt-Rosen *et al.* 2017, Liang *et al.* 2021), providing valuable reference for automatic annotation of scATAC-seq data, which is also known as the label transfer task (You *et al.* 2019, Brbić *et al.* 2020). Transferring labels between scRNA-seq and scATAC-seq data falls into the category of diagonal integration tasks (Argelaguet *et al.* 2021), since scRNA-seq data and scATAC-seq data usually consist of unpaired cells with distinct unmatched features, hence there is no direct correspondence between them. Diagonal integration methods aim to construct a low-dimensional latent space or an integrated count matrix, where the technology-induced differences are removed and cell-identity is preserved from the single-modality dataset (Zhang *et al.* 2022). Based on the integrated cell representations, *k*-nearest neighbors (kNNs) classifier or other classifiers can be applied to transfer labels between modalities. Current diagonal integration methods can be divided into two categories according to their strategies of processing raw omics features. The first category simplifies the diagonal integration into horizontal integration (Argelaguet *et al.* 2021). For example, Seurat (Hao *et al.* 2021), scGCN (Song *et al.* 2021), and scJoint (Lin *et al.* 2022) transform the scATAC-seq features into gene activity matrices (GAM) based on the prior knowledge about regulation relationship between chromatin accessibility and genes (Argelaguet *et al.* 2021, Zhang *et al.* 2022), and then integrates scRNA-seq and scATAC-seq horizontally. The second category directly models on the original omics features. For example, SCIM (Stark *et al.* 2020) and MMD-MA (Liu *et al.* 2019) input raw scATAC-seq features and raw scRNA-seq features into different neural networks, and then employ adversarial training or maximum mean discrepancy minimization to align latent features between modalities.

Both strategies for processing raw omics features have their advantages and constraints. The first strategy can greatly reduce the dimensionality of scATAC-seq features (from hundreds of thousands to tens of thousands), thereby reducing the computational complexity. However, transforming scATAC-seq data may lose part of information of raw data, hence the transformed scATAC-seq can be inaccurate (Argelaguet *et al.* 2021, Zhang *et al.* 2022). The second strategy can preserve the information of raw data and better reflect the relationships between modalities. However, due to lack of prior correspondence, the second strategy can introduce more severe artificial alignment (i.e. over-alignment) than the first strategy (Xu and McCord 2022). GLUE (Cao and Gao 2022) adopts the second strategy, but it incorporates prior knowledge about feature interaction between modalities to learn cellular embeddings. However, whether GLUE’s strategy is good enough to avoid artificial alignment deserves further discussion. Note that the first strategy can map different modalities into the same feature space, it may be a good starting point for diagonal integration to reduce the risk of over-alignment. Nevertheless, feature transformation between modalities may lose information, which can hurt the integration performance.

Here, we present a novel transfer learning method to transfer labels from scRNA-seq data to scATAC-seq data, scNCL, which achieves the state-of-the-art label transfer performance using a neural-network approach. We start from simplifying diagonal integration into horizontal integration by transforming the scATAC-seq feature to GAM using existing tools, such as Signac (Stuart *et al.* 2021), which helps to reduce the risk of over-alignment and computational complexity. However, information loss caused by the feature transformation between modalities would affect integration performance by altering pairwise distance measurements of cells in the raw feature space. To deal with this problem, we introduce neighborhood contrastive learning (NCL) to preserve the neighborhood structure of scATAC-seq cells in the raw feature space. Briefly, we compute a kNN graph for scATAC-seq cells based on the raw chromatin accessibility features, and conserve the kNN graph throughout the feature learning process with contrastive learning, which concentrates embeddings between nearest neighbors and separates embeddings between randomly selected cell pairs (Yan *et al.* 2022, 2023). To make latent features transferable between modalities, we use a new regularization loss for feature projection and a feature alignment (FA) loss to align cellular embeddings between modalities. Experiments on six datasets, including Mouse Cell Atlas and Human Cell Atlas, demonstrate that scNCL outperforms other state-of-the-art methods with respect to transfer accuracy of common cell types and detection of novel [or none-of-the-above, NOTA (Yang *et al.* 2022)] cell types. In addition, we show that scNCL can help refine already labeled scATAC-seq datasets.

## 2 Materials and methods

### 2.1 Overview

scNCL is a semi-supervised framework for cross-modal label transfer, which is motivated by scJoint. Specifically, scNCL learns a feature extractor network: *f* and a classifier network: *g*. The inputs consist of three parts: a gene expression matrix (GEM) from scRNA-seq, a GAM from scATAC-seq, and low-dimensional representations of raw scATAC-seq features (e.g. principal components matrix or tSNE coordinates). Before training, a kNN graph is constructed based on the low-dimensional representations of raw scATAC-seq data. At each training step, two minibatches of cells are sampled from GEM and GAM, respectively. The feature extractor projects these cells into a low-dimensional latent space and then the classifier infers the cell-type assignments for each cell.

**Figure 1.**
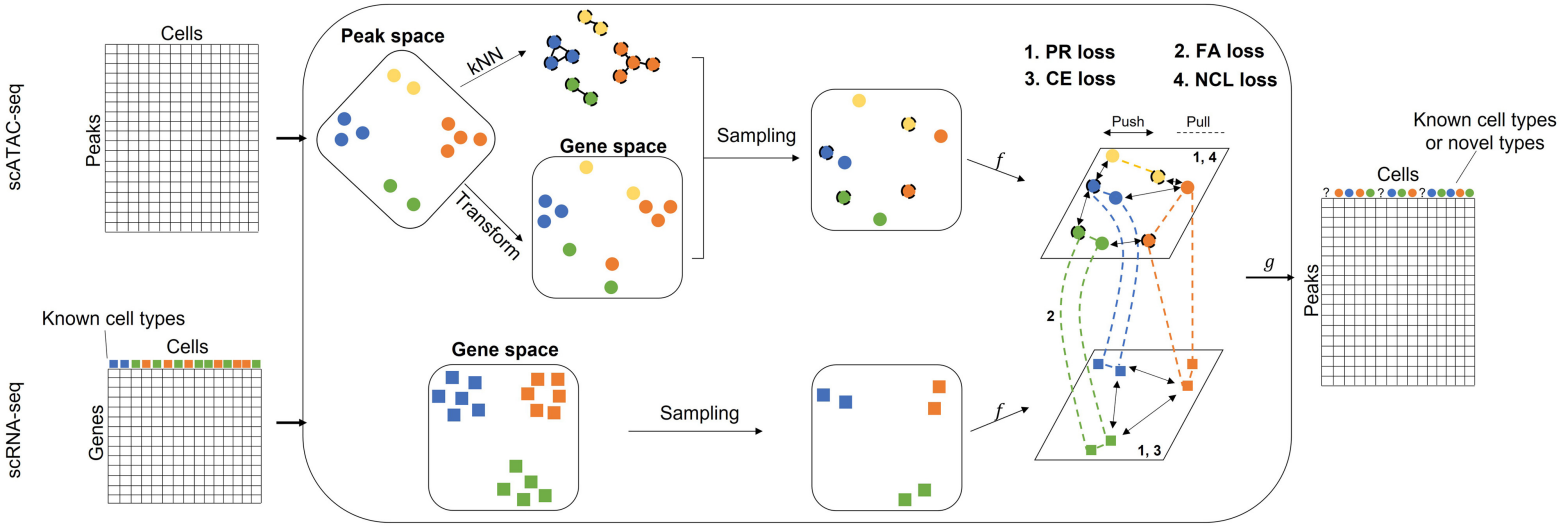
Overview of scNCL. Two minibatches of cells sampled from GEM and GAM are input to the feature extractor. Four loss terms together determine the cellular embeddings in the latent space through pushing dissimilar cells apart and pulling similar cells together. PR and NCL loss influence the embeddings of scATAC-seq cells. PR and CE loss influence scRNA-seq cells. FA loss influence the alignment of cells between two modalities. The classifier network infers cells as known types or as novel types (denoted by “?”) when its prediction confidence of known types is lower than a certain threshold.

To learn the encoder and classifier well, scNCL uses four loss functions: (i) to regularize the whole latent space, a new projection regularization (PR) loss is used; (ii) to explicitly harmonize embeddings between scRNA-seq and scATAC-seq, a FA loss is used; (iii) to learn discriminative features for various cell types, a cross-entropy (CE) loss is used for supervised learning on scRNA-seq data; (iv) to preserve the neighborhood structure of scATAC-seq cells in raw feature space, a NCL loss is used. The PR loss, FA loss, and NCL loss are used to optimize the encoder while the CE loss optimizes the whole network. The overall framework of scNCL is depicted in Fig. 1.

### 2.2 Model

#### 2.2.1 Preliminary

Given two datasets: a labeled scRNA-seq dataset: $D^{l}=\left\{X^{l},Y^{l}\right\}$ and an unlabeled scATAC-seq dataset $D^{u}=\left\{X^{u}\right\}$. $X^{l}={\left[x_{1}^{l},x_{2}^{l},\ldots ,x_{N_{l}}^{l}\right]}^{T}\in R^{N_{l}\times M}$ denotes the GEM and $X^{u}={\left[x_{1}^{u},x_{2}^{u},\ldots ,x_{N_{u}}^{u}\right]}^{T}\in R^{N_{u}\times M}$ denotes the GAM, where $N_{l},N_{u}$ denotes the number of cells in dataset $D^{l}$ and $D^{u}$, respectively, and *M* denotes the number of shared genes between two datasets. $Y^{l}=\left[y_{1},y_{2},\ldots ,y_{N_{l}}\right],y_{i}\in \{1,2,\ldots ,K\}$, where *K* denotes the number of cell types in $D^{l}$. We assume that $D^{u}$ contains cell types that intersect with *K* types in $D^{l}$. We refer all cell types in $D^{l}$ as known types; those shared cell types between $D^{l}$ and $D^{u}$ are referred as common types while those unique cell types in $D^{u}$ are referred as novel types. The goal of label transfer task is not only to precisely infer common types but also to detect/identify novel types (if applicable).

For each cell’s gene expression/activity profile $x^{u}\in D^{u}$ or $x^{l}\in D^{l}$, scNCL takes it as input and the feature extractor *f* parameterized by θ projects the cell into the embedding space: $h^{u}=f(x^{u};\theta ),h^{l}=f(x^{l};\theta ),h^{u},h^{l}\in R^{d}(d\ll M)$, where *d* denotes the embedding dimensionality. Then, the classifier network *g* takes the feature embedding as input and outputs *K*-class probability vector after softmax transformation, r=Softmax(g(h)). The predicted class is defined as $\hat{y}=\underset{k}{\text{argmax}}r(k)$ and the prediction confidence for each cell is defined as $e=\underset{k}{\text{max}}r(k)$, where *k* denotes the *k*-th class. The confidence that a cell is predicted to be a novel type is defined as $\tilde{e}=1-e$. Unless specified, following confidence all refer to *e*.

#### 2.2.2 Projection regularization loss

At each training step, a minibatch of cells is generated by sampling equal-sized subsets of cells from $X^{l}$ and $X^{u}$, $B_{\mathrm{mini}}=B^{l}\cup B^{u}$, where $B^{u}$ and $B^{l}$ contain *N* cells, respectively.

Similar to scJoint, scNCL aims to build an orthogonal embedding space and to maximize the variability of embeddings. To achieve this goal, scNCL uses the PR loss, which is adapted from the NNDR loss (Lin *et al.* 2022). The NNDR loss (Lin *et al.* 2022) is defined as: where *B* denotes $B^{l}$ or $B^{u}$; |B| denotes the number of elements in *B*; $h_{b}$ denotes embedding of cell *b* and $h_{b}(j)$ denotes the *j*-th dimension of embedding; $\bar{h}=\frac{1}{\left|B\right|}\sum_{i\in B}h_{i}$; $A_{ij}$ denotes the element of feature correlation (covariance) matrix of batch *B*. Since $\bar{h}$ denotes the mean of embeddings, minimizing the third term is equivalent to fixing the mean of embeddings near zero. The second term minimizes the correlation between all embedding dimension pairs to achieve orthogonality. The first term maximizes the variability of embeddings. The NNDR loss is applied to $B^{l}$ and $B^{u}$, respectively.


(1) $$\begin{array}{c} L_{\mathrm{NNDR}}(B,\theta )={\left(\frac{1}{|B|\cdot d}\sum_{b\in B}\sum_{j=1}^{d}\left|h_{b}(j)-\bar{h}(j)\right|\right)}^{-1}+\frac{1}{d^{2}}\sum_{i\neq j}\left|A_{ij}\right|+\frac{1}{d}\sum_{j=1}^{d}|\bar{h}(j)|, \end{array}$$

One limitation of the NNDR loss is that it would aggravate the misalignment of embeddings between modalities. More specifically, embeddings of $B^{l}$ are not only affected by the NNDR loss but also affected by the following CE loss, which can further enlarge the variability of embeddings (Liu *et al.* 2020, Cao *et al.* 2021). However, $B^{u}$’s embeddings variability is mainly affected by the NNDR loss. Consequently, when input features between modalities are not perfectly aligned, misalignment between modalities in the embedding space can be enlarged during learning progressing. Therefore, to keep consistent variability growth between modalities, scNCL removes the first term in the NNDR loss for $B^{l}$. Then, the PR loss is defined as: where $L_{\mathrm{NND}R^{-}}^{l}$ denotes the NNDR loss that removes the first term.


(2) $$\begin{array}{c} L_{PR}=L_{\mathrm{NNDR}}^{u}+L_{\mathrm{NND}R^{-}}^{l} \end{array},$$

#### 2.2.3 Feature alignment loss

The FA loss is used to harmonize embeddings between modalities. Briefly, this loss attempts to maximize the cosine similarity between scATAC-seq and scRNA-seq cell pairs (Lin *et al.* 2022). In specific, scNCL first computes the cosine similarity of every cell pair between $B^{u}$ and $B^{l}$ using their embeddings. Those pairs with high similarities may correspond to the same cell type, which should be further aligned. To find those pairs, for each cell $x^{u}\in B^{u}$, scNCL finds the corresponding cell $x^{l}\in B^{l}$ that maximizes cosine similarity $\text{cos}\;\left(h^{u},h^{l}\right)$. Then, scNCL takes the top *p* fraction of cells with the highest similarity scores from $B^{u}$ to compute the FA loss: where $F_{p}$ denotes a subset of cells from $B^{u}$ with top similarity scores; $\left|F_{p}\right|$ denotes the number of elements in $F_{p}$; *i* denotes the index of cell in $B^{l}$ that maximize $\text{cos}\;\left(h_{b}^{u},h_{i}^{l}\right)$.


(3) $$\begin{array}{c} L_{FA}\left(B^{u}\right)=-\frac{1}{\left|F_{p}\right|}\sum_{b\in F_{p}}\;\text{cos}\;\left(h_{b}^{u},h_{i}^{l}\right) \end{array},$$

#### 2.2.4 Cross entropy loss

To learn discriminative features for various cell types, scNCL adopts the CE loss as a signal to supervise the cell-type learning on $D^{l}$: where $\left|B^{l}\right|$ denotes the number of elements in $B^{l}$; $y_{b}^{l}$ denotes the cell-type label of cell *b*; and $r_{b}(k)$ denotes the *k*-th dimension of $r_{b}$.


(4) $$\begin{array}{c} L_{CE}=-\frac{1}{\left|B^{l}\right|}\sum_{b\in B^{l}}\sum_{k=1}^{K}1\left(y_{b}^{l}=k\right)\cdot \;\text{log}\;\left(r_{b}(k)\right) \end{array},$$

#### 2.2.5 Neighborhood contrastive learning loss

As mentioned above, mapping scATAC-seq data to GAM may result in information loss, leading to degradation of label transfer performance. Concretely, several problems may be encountered: (i) some scATAC-seq cells with the same type become more distant from each other in the transformed gene space compared to the raw feature space. These cells can be further separated in the latent space. After alignment with scRNA-seq data, these scATAC-seq cells may be classified as different types; (ii) some scATAC-seq cells with different types become closer in the transformed gene space compared to the raw feature space. These cells can stay closer in the latent space. After alignment with scRNA-seq data, these scATAC-seq cells with different types may be classified as the same type. Moreover, the cell-type supervision is only posed for known cell types (in scRNA-seq), which indicates the model learns discriminative representations faster on the known types compared to the novel types (in scATAC-seq data). This leads to smaller intra-class invariance of known types compared to novel types (Cao *et al.* 2021). Consequently, it is hard to distinguish novel types from known types. In conclusion, changes in cell–cell distance relationship caused by modality transformation and feature projection can hinder accurate identification of cell types for scATAC-seq data.

To address above problems, scNCL employs contrastive learning to regularize the changes in cell–cell distance relationship. scNCL does not maintain the distances between every pair of cells unchanged instead it builds a neighborhood graph among all scATAC-seq cells and preserves the neighborhood graph, which is more robust and more effective. Specifically, a kNN graph with neighborhood size $k_{0}$ is built based on the raw scATAC-seq features. For each cell in minibatch $x^{u}\in B^{u}$, one of their neighbors is sampled from their neighbor sets, forming a new minibatch, $B_{nn}^{u}=\left\{{\hat{x}}_{1}^{u},\ldots ,{\hat{x}}_{N}^{u}\right\}$ and a minibatch of positive pairs $\left\{\left(x_{1}^{u},{\hat{x}}_{1}^{u}\right),\ldots ,\left(x_{N}^{u},{\hat{x}}_{N}^{u}\right)\right\}$. The NCL loss can be written as: where ${\hat{h}}_{b}^{u}=f\left({\hat{x}}_{b}^{u};\theta \right)$; $\sigma \left(h_{b}^{u},{\hat{h}}_{b}^{u}\right)=\text{exp}\;\left(\frac{\langle h_{b}^{u},{\hat{h}}_{b}^{u}\rangle }{\tau \left\|h_{b}^{u}\right\|\cdot \left\|{\hat{h}}_{b}^{u}\right\|}\right)$, where $\langle h_{b}^{u},{\hat{h}}_{b}^{u}\rangle$ denotes inner product of two vectors and $\left\|h_{b}^{u}\right\|$ denotes the *L*2-norm of a vector; τ is a positive constant. To minimize $L_{\mathrm{NCL}}$, we maximize the embeddings’ similarities between cells and their neighbors, and minimize the embeddings’ similarities between cells and their non-neighbors. Overall, the training loss function is defined as: where $\lambda_{1}$ and $\lambda_{2}$ denote the weights of different loss terms.


(5) $$\begin{array}{c} \begin{array}{c} L_{\mathrm{NCL}}=-\frac{1}{2N}\sum_{b=1}^{N}\;\text{log}\;\left(\frac{\sigma \left(h_{b}^{u},{\hat{h}}_{b}^{u}\right)}{\sum_{r=1}^{N}\sigma \left(h_{b}^{u},{\hat{h}}_{r}^{u}\right)+\sum_{r=1}^{N}1(r\neq b)\cdot \sigma \left(h_{b}^{u},h_{r}^{u}\right)}\right)\\ +\text{log}\;\left(\frac{\sigma \left({\hat{h}}_{b}^{u},h_{b}^{u}\right)}{\sum_{r=1}^{N}\sigma \left({\hat{h}}_{b}^{u},h_{r}^{u}\right)+\sum_{r=1}^{N}1(r\neq b)\cdot \sigma \left({\hat{h}}_{b}^{u},{\hat{h}}_{r}^{u}\right)}\right), \end{array} \end{array}$$

(6) $$\begin{array}{c} L=L_{CE}+0.1L_{PR}+\lambda_{1}L_{FA}+\lambda_{2}L_{\mathrm{NCL}} \end{array},$$

## 3 Results

### 3.1 Datasets

We collected six datasets and grouped them into four datasets: one is a paired dataset and three are unpaired datasets. Specifically, the paired dataset is a publicly available human PBMC “multiome” (granulocyte-sorted 10k, P 2020) dataset from 10X Genomics, which profiles gene expression and chromatin accessibility in the same cell. We treated this dataset as originating from two different experiments. For the unpaired datasets, one of them is obtained from a T cell stimulation experiment (Mimitou *et al.* 2021, Lin *et al.* 2022), which consists of data generated by CITE-seq and data generated by ASAP-seq. CITE-seq profiles the gene expression with surface protein in the same cell, and ASAP-seq profiles the chromatin accessibility with surface protein in the same cell. Another unpaired dataset consists of two mouse cell atlases, including the FACS-based data from Tabula Muris atlas (Schaum *et al.* 2018) for scRNA-seq and the atlas from Cusanovich *et al.* (2018) for scATAC-seq data. The last dataset consists of two human cell atlases, including scRNA-seq data from human fetal samples (Cao *et al.* 2020) and scATAC-seq data from human fetal tissues (Domcke *et al.* 2020).

For the unpaired dataset containing two mouse cell atlases, we used cell-type annotations provided by Lin *et al.* (2022) for all cells to ensure that the naming convention is consistent. The GAM for scATAC-seq data was obtained from the original study (Cusanovich *et al.* 2018). For the unpaired dataset containing two human cell atlases, the scRNA-seq atlas was subsampled as Lin *et al.* (2022) to construct a balanced training set by subsampling $\max\left\{0.05n_{i},10\;000\right\}$ cells for cell type *i* with number of cells $n_{i}>10\;000$. The GAM for scATAC-seq data was from the original study (Domcke *et al.* 2020). For the paired dataset, Signac was used to generate the GAM for scATAC-seq data and Seurat’s annotations were used as ground truth of cell types. For convenience, we referred the unpaired dataset containing two mouse cell atlases as MCA dataset, the unpaired dataset containing two human cell atlases as HFA dataset, the unpaired dataset containing CITE-seq data and ASAP-seq data as CITE-ASAP dataset, and the paired dataset as PBMC dataset.

### 3.2 Evaluation of label transfer performance

We evaluated the performance of label transfer from two aspects: (i) prediction accuracy of common cell types. The overall accuracy rate was computed for the common cell types between scRNA-seq and scATAC-seq data. It is defined as: where $D_{\mathrm{com}}^{u}$ denotes the subset of cells in $D^{u}$ with common cell types; $\left|D_{\mathrm{com}}^{u}\right|$ denotes the number of cells in $D_{\mathrm{com}}^{u}$. We also computed cell-type classification *F*1 score to inspect the prediction performance of specific cell types. The *F*1 score is harmonic mean of precision and recall for each cell type *i*: where ${\text{TP}}_{i}$ denotes the number of cells that are correctly predicted as type *i*; ${\text{FP}}_{i}$ denotes the number of cells that are incorrectly predicted as type *i*; ${\text{FN}}_{i}$ denotes the number of cells that belong to type *i* but predicted as other types. (ii) Novel-type detection performance. Since identification of novel types is a binary classification problem, we reported the threshold-free area under the receiver-operator curve (AUROC) using the prediction confidence of novel types, $\tilde{e}$. In this context, the prediction target of cells belonging to novel types is one while the others’ is zero. To balance these two aspects, we followed Dhamija *et al.* (2018) to report the Open-set Classification Rate (OSCR), which measures the trade-off between accuracy and novel-type detection rate as a threshold on the confidence of the predicted class is varied (Dhamija *et al.* 2018, Vaze *et al.* 2021).


(7) $$\begin{array}{c} \;\text{Accuracy}=\frac{\sum_{i\in D_{\mathrm{com}}^{u}}1\left(y_{i}={\hat{y}}_{i}\right)}{\left|D_{\mathrm{com}}^{u}\right|} \end{array},$$

(8) $$\begin{array}{c} F1_{i}=\frac{2\times {\text{Precision}}_{i}\times {\text{Recall}}_{i}}{{\text{Precision}}_{i}+{\text{Recall}}_{i}},\\ {\text{Precision}}_{i}=\frac{{\text{TP}}_{i}}{{\text{TP}}_{i}+{\text{FP}}_{i}},{\text{Recall}}_{i}=\frac{{\text{TP}}_{i}}{{\text{TP}}_{i}+{\text{FN}}_{i}}, \end{array}$$

### 3.3 scNCL can infer common cell types accurately and robustly

We first evaluated scNCL’s performance in scenarios where scRNA-seq and scATAC-seq data have the same collections of cell types. We used the PBMC dataset, a subset of MCA dataset (referred as MCA-subset), and multiple subsets of HFA datasets. Specifically, we extracted 19 common cell types from MCA dataset to focus on transferring common cell types, resulting in 19 726 cells for scRNA-seq and 57 563 cells for scATAC-seq. Also, we extracted 54 common types from HFA dataset, resulting in 433 695 cells for scRNA-seq and 656 074 cells for scATAC-seq (referred as HFA-subset-full). In addition, to evaluate the robustness to various dataset sizes, we subsampled the HFA-subset-full dataset with various number of cells: 20 000 for scRNA-seq and 30 000 for scATAC-seq, 40 000 for scRNA-seq and 60 000 for scATAC-seq, 80 000 for scRNA-seq and 120 000 for scATAC-seq, and 160 000 for scRNA-seq and 240 000 for scATAC-seq, all of which were referred as HFA-subset-50k/100k/200k/400k, respectively. We repeated the sampling five times for HFA-subset-50k/100k/200k and repeated three times for HFA-subset-400k. All these datasets have the same collections of cell types between scRNA-seq and scATAC-seq. Seven state-of-the-art data integration methods for single-cell data were used for comparison: Seurat 4 (Hao *et al.* 2021), scGCN (Song *et al.* 2021), scNym (Kimmel and Kelley 2021), Portal (Zhao *et al.* 2022), Concerto (Yang *et al.* 2022), scJoint (Lin *et al.* 2022), and GLUE (Cao and Gao 2022). Although some of them were originally designed for horizontal integration, they can be extended to integrate scRNA-seq and scATAC-seq by converting the scATAC-seq data to GAM. Detailed settings used for all methods are shown in Supplementary Note A.

**Figure 2.**
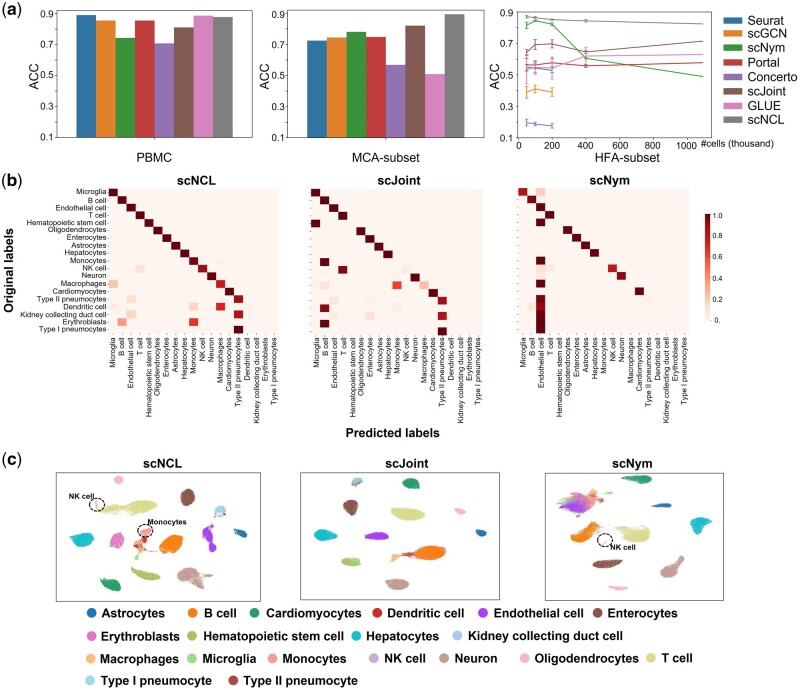
Benchmarking results for common-type transfer. (a) Overall accuracy rate of Seurat, scGCN, scNym, Portal, Concerto, scJoint, GLUE, and scNCL on PBMC, MCA-subset, and HFA-susbets. The right panel shows the line plot of accuracy with standard deviation bar on each side of the mean. (b) (row normalized) Heatmaps comparing the original labels and the transferred labels from scNCL, scJoint, and scNym. Cell types are sorted bottom-up (right-left) according to their percentage in the reference (scRNA-seq) data. Microglia accounts for the highest percentage in reference data. (c) UMAP visualizations of embeddings for scATAC-seq data from scNCL, scJoint, and scNym. Cells are colored by their cell-type annotations.

The assessment results are shown in Fig. 2. On the PBMC dataset, all methods achieve an accuracy rate of 0.7 or higher (Fig. 2a), probably due to the fact of small dataset size and identical cell-type compositions. Seurat achieves the highest accuracy, 0.89. GLUE’s and scNCL’s accuracy are very close to Seurat. For the more complex MCA-subset dataset, in which the heterogeneity of tissues and imbalanced cell types poses substantial challenges to label transfer, all methods except for scNCL, scJoint, and scNym show clear performance drop compared to PBMC dataset. However, scNCL achieves the highest accuracy rate of 0.89 and scJoint achieves the second highest accuracy rate of 0.82. Looking closer at the performance for each cell type, scNCL not only achieves high classification *F*1-scores for major cell types in scRNA-seq but also achieves high *F*1-scores for those minor types in scRNA-seq (Fig. 2b and Supplementary Figs S1–S3). For instance, monocytes account for 2% of scRNA-seq data. scNCL achieves a *F*1-score of 0.52 for monocytes in scATAC-seq while scJoint’s *F*1-score is 0.01. NK cells account for the 1.7% of scRNA-seq data. scNCL achieves a *F*1-score of 0.91 for NK cells in scATAC-seq data while scJoint’s *F*1-score is 0.09. An inspection of UMAP plots also shows that scNCL retains clear clusters in the embedding space for monocytes and NK cells, whereas scJoint mixes them with other major types (Fig. 2c and Supplementary Figs S4 and S5). For the HFA-subset-50k/-100k/-200k/-400k/-full datasets, in which heterogeneity of tissues and highly unbalanced cell-type compositions pose great computational challenges, scNCL’s overall accuracy still maintains a high level while other methods show low accuracy, performance drop with increasing dataset size, or fail with memory error (Seurat, scGCN, and Concerto) (Fig. 2a). In addition, scNCL is computationally efficient and can be easily scaled to million-scale datasets (Supplementary Fig. S6).

Together, these results suggest scNCL is not only robust and accurate to handle various scenarios of label transfer task but also has superior scalability.

### 3.4 scNCL can robustly detect novel types

**Figure 3.**
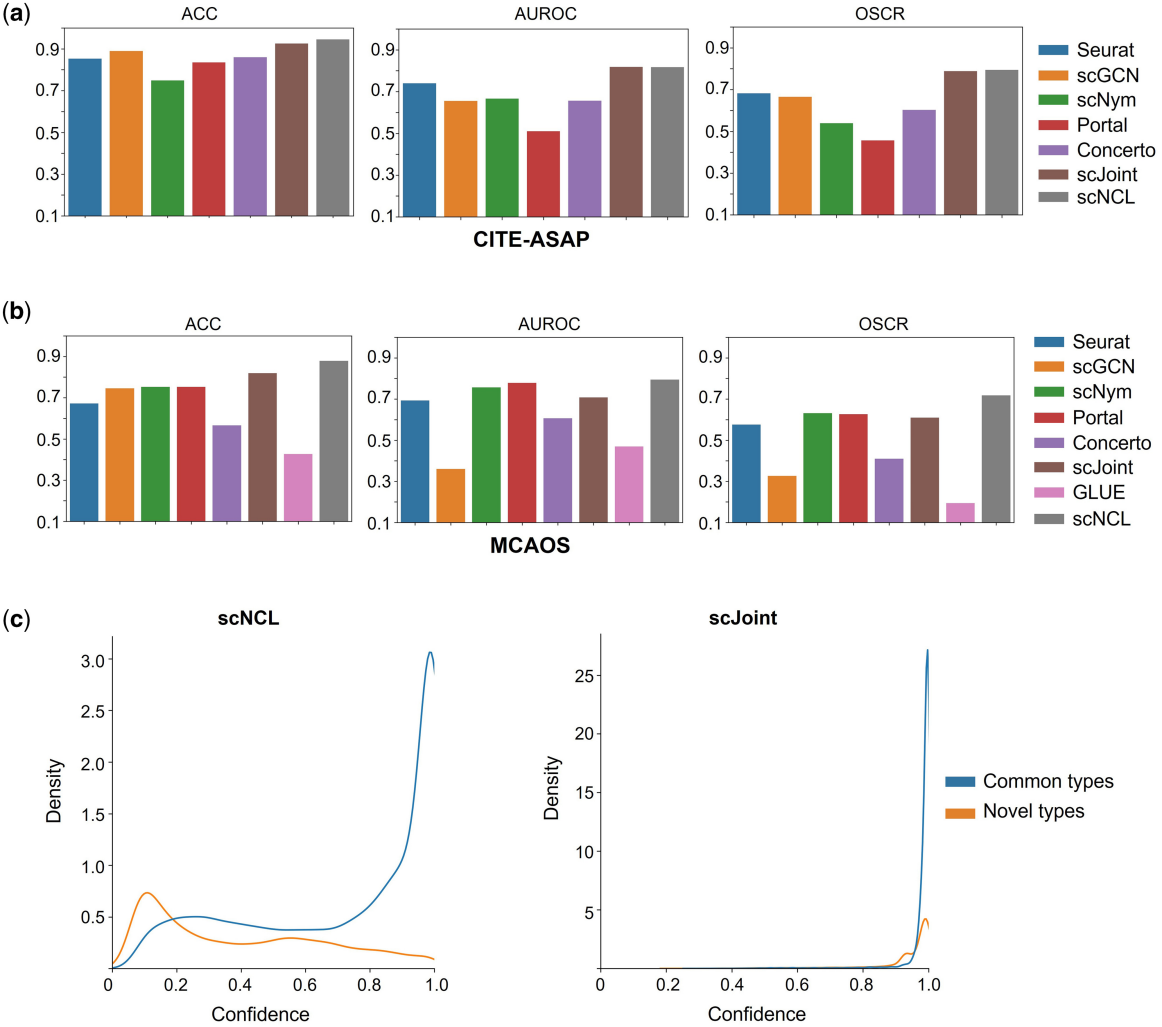
Benchmarking results for common-type transfer and novel-type detection. (a) Overall accuracy, AUROC, and OSCR of Seurat, scGCN, scNym, Portal, Concerto, scJoint, GLUE, and scNCL on CITE-ASAP dataset. (b) Overall accuracy, AUROC, and OSCR of Seurat, scGCN, scNym, Portal, Concerto, scJoint, GLUE, and scNCL on MCAOS dataset. (c) KDE plot for visualizing the distribution of prediction confidence for MCAOS scATAC-seq cells, based on scNCL and scJoint’s outputs.

In many label transfer tasks, the reference data may not cover all of the cell types present in the target data [i.e. category shift (Xu *et al.* 2018)]. So, transfer learning methods should not only precisely infer common cell types but also help to distinguish novel types present in the target data. We first used CITE-ASAP dataset to evaluate scNCL’s performance in identification of novel types. This dataset contains 4502 cells for CITE-seq and 4644 cells for ASAP-seq, with seven cell types and nine cell types, respectively. ASAP-seq data has seven cell types overlapped with CITE-seq data. We applied scNCL to transfer labels from CITE-seq to ASAP-seq. Six methods were included for comparison: Seurat 4, scGCN, scNym, Portal, Concerto, and scJoint. The reason why GLUE was not included is because CITE-seq and ASAP-seq data both consist of two omics while GLUE cannot handle this situation. Detailed settings for compared methods are shown in Supplementary Note A. Results show that scNCL achieves very similar performance with scJoint since their differences on three metrics are small (Fig. 3a). scNCL achieves the highest OSCR and scJoint achieves the second highest. Although other methods can achieve an overall accuracy of 0.8 or higher, they do not perform well enough for novel-type detection on this dataset.

Next, we compared scNCL with other methods in a more complex scenario, in which the category shift between reference and target dataset is more significant. Specifically, we extracted 19 common types of cells from scRNA-seq data in the MCA dataset as reference and used all cells from scATAC-seq data in the MCA dataset as target, resulting in 19 726 scRNA-seq cells and 81 173 scATAC-seq cells (referred as MCAOS dataset). The target data contains 19 common types and 10 novel types. Results show that scNCL delivers the highest overall accuracy rate and AUROC among all methods, leading to the best OSCR value (Fig. 3b). Portal and scNym both achieve high AUROC while they compromise to overall accuracy of common types. scJoint achieves the second highest transfer accuracy but its AUROC is relatively lower than scNCL, Portal, and scNym. To compare the performance between scJoint and scNCL with respect to novel-type detection more intuitively, we visualized the distribution of their prediction confidence, *e* for scATAC-seq cells by kernel density estimation. Figure 3c shows that scNCL’s prediction confidence for cells belonging to common types is mainly concentrated around one while confidence for cells belonging to novel types is mainly concentrated around zero, meaning that it is easy to distinguish novel cell types from common types based on scNCL’s prediction confidence. However, scJoint’s prediction confidence for all cells is concentrated around one, meaning that it is more difficult to distinguish novel types from common types based on its prediction confidence. An inspection of UMAP plot also shows that scNCL embeds cells of novel types into distinct clusters from common types, thereby achieving low prediction confidence for novel-type cells (Supplementary Fig. S7).

Together, these results suggest that scNCL can achieve superior trade-off between inferring common cell types and detection of novel types.

### 3.5 scNCL can help refine scATAC-seq annotations

We showcased that scNCL can be used to refine annotations of already labeled scATAC-seq datasets. Taking MCA dataset as an example, we attempted to apply scNCL to transfer labels from scRNA-seq data to scATAC-seq data. The full scRNA-seq data contains 67 type annotations, and the full scATAC-seq data contains 29 original type annotations. The transferred labels and original labels are plotted in Supplementary Fig. S8. We chose tSNE as the dimensionality reduction tool because we found that in this dataset, separation boundaries are clearer in tSNE plot than in UMAP plot (Supplementary Fig. S9). The tSNE coordinates of scATAC-seq data were obtained from original study (Cusanovich *et al.* 2018).

**Figure 4.**
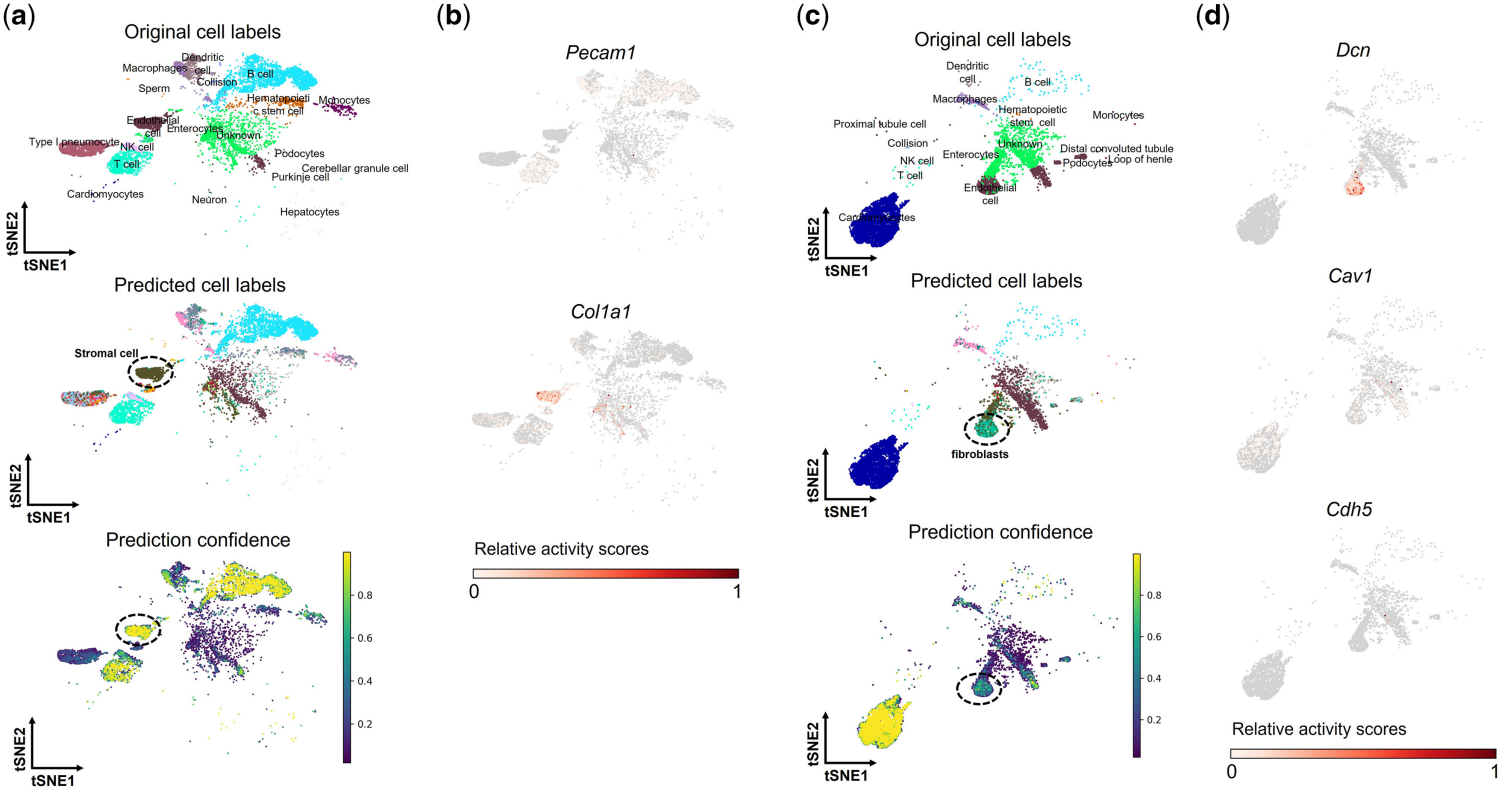
Analysis of scNCL’s predictions for lung and heart cells from scATAC-seq data in MCA dataset. (a) Original labels, predicted labels, and prediction confidence of lung cells. (b**)** Marker expression of endothelial cells in lung (*Pecam1*) and marker expression of stromal cells in lung (*Col1a1*). (c) Original labels, predicted labels, and prediction confidence of heart cells. (d) Marker expression of endothelial cells in heart (*Cav1*, *Cdh5*) and marker expression of fibroblast in heart (*Dcn*).

We find that scNCL annotates a group of cells from lung (originally labeled as “endothelials”) as “stromal cells” (719 cells) with high average confidence (>0.85) (Fig. 4a). These cells show high expression levels of *Col1a1*, which has a high gene expression enrichment in “stromal cells” of lung FACS data (Schaum *et al.* 2018), and show low expression levels of *Pecam1*, which has a high gene expression enrichment in “endothelial” cells of lung FACS data (Schaum *et al.* 2018) (Fig. 4b). Hence, scNCL’s annotations for “stromal cells” are consistent with marker expression levels. The gene ontology (GO) analysis of biological processes using EnrichR (Kuleshov *et al.* 2016, Xie *et al.* 2021) shows that the upregulated genes in scNCL’s refined “stromal cells” are enriched for terms related to extracellular matrix organization, skeletal system development, and inflammatory response (Supplementary Fig. S10), which have been proved by previous studies (Gattazzo *et al.* 2014, Grayson *et al.* 2015, Khoury *et al.* 2020). Furthermore, EnrichR shows that those upregulated genes are enriched in “stromal cells” type in different tissues from Tabula Muris Atlas (Schaum *et al.* 2018). In addition, we find that scNCL annotates a group of cells from heart (originally labeled as “endothelials”) as “fibroblast” (268 cells) with high average confidence (>0.6) (Fig. 4c). These cells show high expression level of *Dcn*, which has a high expression enrichment in “fibroblast” of heart FACS data (Schaum *et al.* 2018), and show low expression level of *Cav1* and *Cdh5*, both of which have high gene expression enrichment in “endothelial” cells of heart FACS data (Schaum *et al.* 2018) (Fig. 4d). The GO analysis of biological processes using EnrichR confirms that the upregulated genes in scNCL’s refined “fibroblast” are enriched for terms related to fibroblast growth factor receptor signaling pathway and pinocytosis (Steinman *et al.* 1974), and are enriched in “fibroblast” type in different tissues from Tabula Muris Atlas (Supplementary Fig. S11).

As another example, we applied scNCL to transfer labels from scRNA-seq data in the HFA dataset to scATAC-seq data in the HFA dataset. The scRNA-seq data contains 77 cell-type annotations and scATAC-seq data contains 54 original type annotations. The transferred labels and original labels are shown in Supplementary Fig. S12. We find that most of scNCL’s annotations are consistent with the original labels. Interestingly, scNCL annotates a small cluster of cells from Cerebrum (originally labeled as “vascular endothelial cells”) as “microglia” (214 cells), which do not appear in the original label set (Supplementary Fig. S13a). These cells show high expression level of *Cyth4*, *Spp1*, and *Olr1*, all of which are markers of “microglia” cells in scRNA-seq data (Cao *et al.* 2020) (Supplementary Fig. S13b). The GO analysis of biological processes using EnrichR confirms that the upregulated genes in scNCL’s refined “microglia” are enriched for terms related to peripheral nervous system neuron development (Kabba *et al.* 2018) and nerve growth factor signaling pathway (Sofroniew *et al.* 2001), and are enriched in “microglia” type in different tissues from Descartes Atlas (Cao *et al.* 2020) (Supplementary Fig. S14). The possible reason why “microglia” was not detected in the original study is that “microglia” is a rare type in the scRNA-seq data and their adopted non-negative least squares-based label transfer strategy failed to transfer it (Domcke *et al.* 2020). This finding indicates that scNCL can help recover rare cell types that have not been identified in those labeled scATAC-seq datasets.

## 4 Discussion

In this work, we present scNCL, an automated cell-type identification method that utilizes the well-annotated scRNA-seq data to annotate scATAC-seq data. To deal with the heterogeneous features between modalities, we propose to transform scATAC-seq features to gene activity scores with the prior knowledge and introduce contrastive learning to preserve the neighborhood structure of cells in raw scATAC-seq data. Multiple loss functions are used to achieve structure preservation and transferable latent features.

Experiments on various datasets demonstrate that scNCL achieves higher transfer accuracy of common cell types and better detection of novel types compared to existing horizontal integration methods and diagonal integration methods. We also showcase that scNCL can be applied to refine annotations of labeled scATAC-seq data. In addition, benefiting from the efficient architecture, scNCL is fast and scalable to million-scale datasets. Finally, ablation studies demonstrate the rationality of our PR loss over original NNDR loss and the superiority of our proposed NCL loss (Supplementary Note B1). Parameter sensitivity experiments also suggest that scNCL is generally robust to the choice of hyperparameters (Supplementary Note B2).

In this study, we only focus on transferring labels from scRNA-seq to scATAC-seq. However, in principle, scNCL can be extended to transfer information across other modalities if the input can be transformed into the same feature space, such as scRNA-seq to methylation data (Lin *et al.* 2022). Despite of the superior performance of scNCL, there still exist some directions for future improvement. For example, scNCL relies on pre-defined GAM to bridge the gap of heterogeneous features between scRNA-seq and scATAC-seq, which may fail in complex scenarios (Argelaguet *et al.* 2021). Although our proposed NCL loss can regularize cell–cell distance relationship in raw scATAC-seq data, it only provides a complementary signal for feature learning and therefore cannot dominate the alignment between scRNA-seq and scATAC-seq, which is crucial for label transfer. One possible solution is to incorporate the process of transforming scATAC-seq feature to gene activity scores into model training, such as scDart (Zhang *et al.* 2022).

## Supplementary Material

btad505_Supplementary_Data

## References

[btad505-B1] Argelaguet R , Cuomo AS, Stegle O et al Computational principles and challenges in single-cell data integration. Nat Biotechnol 2021;39:1202–15. 33941931 10.1038/s41587-021-00895-7

[btad505-B2] Brbić M , Zitnik M, Wang S et al MARS: discovering novel cell types across heterogeneous single-cell experiments. Nat Methods 2020;17:1200–6. 33077966 10.1038/s41592-020-00979-3

[btad505-B3] Cao J , O’Day DR, Pliner HA et al A human cell atlas of fetal gene expression. Science 2020;370:eaba7721. 33184181 10.1126/science.aba7721 PMC7780123

[btad505-B4] Cao K , Brbic M, Leskovec J. Open-world semi-supervised learning. arXiv, 2021, preprint: not peer reviewed. 10.48550/arXiv.2102.03526.

[btad505-B5] Cao Z-J , Gao G. Multi-omics single-cell data integration and regulatory inference with graph-linked embedding. Nat Biotechnol 2022;40:1458–66. 35501393 10.1038/s41587-022-01284-4 PMC9546775

[btad505-B6] Chen H , Lareau C, Andreani T et al Assessment of computational methods for the analysis of single-cell ATAC-seq data. Genome Biol 2019;20:241. 31739806 10.1186/s13059-019-1854-5 PMC6859644

[btad505-B7] Cusanovich DA , Hill AJ, Aghamirzaie D et al A single-cell atlas of in vivo mammalian chromatin accessibility. Cell 2018;174:1309–24.e18. 30078704 10.1016/j.cell.2018.06.052 PMC6158300

[btad505-B8] Dhamija AR , Günther M, Boult T. Reducing network agnostophobia. Advances in Neural Information Processing Systems. Vol. 31. 2018.

[btad505-B9] Domcke S , Hill AJ, Daza RM et al A human cell atlas of fetal chromatin accessibility. Science 2020;370:eaba7612. 33184180 10.1126/science.aba7612 PMC7785298

[btad505-B10] Gattazzo F , Urciuolo A, Bonaldo P. Extracellular matrix: a dynamic microenvironment for stem cell niche. Biochim Biophys Acta 2014;1840:2506–19. 24418517 10.1016/j.bbagen.2014.01.010 PMC4081568

[btad505-B11] granulocyte-sorted 10k, P. Single cell multiome ATAC gene expression demonstration data by cell ranger arc 1.0.0. 10x Genomics. 2020. https://support.10xgenomics.com/single-cell-multiome-atac-gex/datasets/1.0.0/pbmc_granulocyte_sorted_10k.

[btad505-B12] Grayson WL , Bunnell BA, Martin E et al Stromal cells and stem cells in clinical bone regeneration. Nat Rev Endocrinol 2015;11:140–50. 25560703 10.1038/nrendo.2014.234 PMC4338988

[btad505-B13] Grosselin K , Durand A, Marsolier J et al High-throughput single-cell chip-seq identifies heterogeneity of chromatin states in breast cancer. Nat Genet 2019;51:1060–6. 31152164 10.1038/s41588-019-0424-9

[btad505-B14] Hao Y , Hao S, Andersen-Nissen E et al Integrated analysis of multimodal single-cell data. Cell 2021;184:3573–87.e29. 34062119 10.1016/j.cell.2021.04.048 PMC8238499

[btad505-B15] Kabba JA , Xu Y, Christian H et al Microglia: housekeeper of the central nervous system. Cell Mol Neurobiol 2018;38:53–71. 28534246 10.1007/s10571-017-0504-2 PMC11481884

[btad505-B16] Khoury O , Atala A, Murphy SV. Stromal cells from perinatal and adult sources modulate the inflammatory immune response in vitro by decreasing Th1 cell proliferation and cytokine secretion. Stem Cells Transl Med 2020;9:61–73. 31638323 10.1002/sctm.19-0123 PMC6954711

[btad505-B17] Kimmel JC , Kelley DR. Semisupervised adversarial neural networks for single-cell classification. Genome Res 2021;31:1781–93. 33627475 10.1101/gr.268581.120 PMC8494222

[btad505-B18] Kuleshov MV , Jones MR, Rouillard AD et al Enrichr: a comprehensive gene set enrichment analysis web server 2016 update. Nucleic Acids Res 2016;44:W90–7. 27141961 10.1093/nar/gkw377 PMC4987924

[btad505-B19] Liang Z , Li M, Zheng R et al SSRE: cell type detection based on sparse subspace representation and similarity enhancement. Genomics Proteomics Bioinformatics 2021;19:282–91. 33647482 10.1016/j.gpb.2020.09.004 PMC8602764

[btad505-B20] Lin Y , Wu T-Y, Wan S et al ScJoint integrates atlas-scale single-cell RNA-seq and ATAC-seq data with transfer learning. Nat Biotechnol 2022;40:703–10. 35058621 10.1038/s41587-021-01161-6 PMC9186323

[btad505-B21] Liu B , Cao Y, Lin Y et al Negative margin matters: understanding margin in few-shot classification. In: Computer Vision–ECCV 2020: 16th European Conference, Glasgow, UK, August 23–28, 2020, Proceedings, Part IV 16. 438–55. Springer, 2020.

[btad505-B22] Liu J , Huang Y, Singh R et al Jointly embedding multiple single-cell omics measurements. Algorithms Bioinform 2019;143:10. 34632462 10.4230/LIPIcs.WABI.2019.10 PMC8496402

[btad505-B23] Mimitou EP , Lareau CA, Chen KY et al Scalable, multimodal profiling of chromatin accessibility, gene expression and protein levels in single cells. Nat Biotechnol 2021;39:1246–58. 34083792 10.1038/s41587-021-00927-2 PMC8763625

[btad505-B24] Qiu X , Mao Q, Tang Y et al Reversed graph embedding resolves complex single-cell trajectories. Nat Methods 2017;14:979–82. 28825705 10.1038/nmeth.4402 PMC5764547

[btad505-B25] Rozenblatt-Rosen O , Stubbington MJ, Regev A et al The human cell atlas: from vision to reality. Nature 2017;550:451–3. 29072289 10.1038/550451a

[btad505-B26] Schaum N , Karkanias J, Neff NF et al Single-cell transcriptomics of 20 mouse organs creates a Tabula Muris. Nature 2018;562:367. 30283141 10.1038/s41586-018-0590-4 PMC6642641

[btad505-B27] Sofroniew MV , Howe CL, Mobley WC. Nerve growth factor signaling, neuroprotection, and neural repair. Annu Rev Neurosci 2001;24:1217–81. 11520933 10.1146/annurev.neuro.24.1.1217

[btad505-B28] Song Q , Su J, Zhang W. scGCN is a graph convolutional networks algorithm for knowledge transfer in single cell omics. Nat Commun 2021;12:3826. 34158507 10.1038/s41467-021-24172-y PMC8219725

[btad505-B29] Stark SG , Ficek J, Locatello F et al; Tumor Profiler Consortium. SCIM: universal single-cell matching with unpaired feature sets. Bioinformatics 2020;36:i919–27. 33381818 10.1093/bioinformatics/btaa843 PMC7773480

[btad505-B30] Steinman RM , Silver JM, Cohn ZA. PINOCYTOSIS in FIBROBLASTS: quantitative studies in vitro. J Cell Biol 1974;63:949–69. 4140194 10.1083/jcb.63.3.949 PMC2109356

[btad505-B31] Stuart T , Srivastava A, Madad S et al Single-cell chromatin state analysis with Signac. Nat Methods 2021;18:1333–41. 34725479 10.1038/s41592-021-01282-5 PMC9255697

[btad505-B32] Treutlein B , Brownfield DG, Wu AR et al Reconstructing lineage hierarchies of the distal lung epithelium using single-cell RNA-seq. Nature 2014;509:371–5. 24739965 10.1038/nature13173 PMC4145853

[btad505-B33] Vaze S , Han K, Vedaldi A et al Open-set recognition: a good closed-set classifier is all you need. arXiv, 2021, preprint: not peer reviewed. 10.48550/arXiv.2110.06207.

[btad505-B34] Xie Z , Bailey A, Kuleshov MV et al Gene set knowledge discovery with Enrichr. Curr Protoc 2021;1:e90. 33780170 10.1002/cpz1.90 PMC8152575

[btad505-B35] Xu R , Chen Z, Zuo W et al Deep cocktail network: multi-source unsupervised domain adaptation with category shift. In: *Proceedings of the IEEE Conference on Computer Vision and Pattern Recognition, Salt Lake City, Utah, USA*. June 2018, 3964–73.

[btad505-B36] Xu Y , McCord RP. Diagonal integration of multimodal single-cell data: potential pitfalls and paths forward. Nat Commun 2022;13:3505. 35717437 10.1038/s41467-022-31104-x PMC9206644

[btad505-B37] Yan X , Zheng R, Li M. Globe: a contrastive learning-based framework for integrating single-cell transcriptome datasets. Brief Bioinform 2022;23:bbac311. 35901449 10.1093/bib/bbac311

[btad505-B38] Yan X , Zheng R, Wu F et al CLAIRE: contrastive learning-based batch correction framework for better balance between batch mixing and preservation of cellular heterogeneity. Bioinformatics 2023;39:btad099. 36821425 10.1093/bioinformatics/btad099 PMC9985174

[btad505-B39] Yang M , Yang Y, Xie C et al Contrastive learning enables rapid mapping to multimodal single-cell atlas of multimillion scale. Nat Mach Intell 2022;4:696–709.

[btad505-B40] You K , Long M, Cao Z et al Universal domain adaptation. In: *Proceedings of the IEEE/CVF Conference on Computer Vision and Pattern Recognition, Long Beach, California, USA*. June 2019, 2720–9.

[btad505-B41] Yu W , Uzun Y, Zhu Q et al scATAC-pro: a comprehensive workbench for single-cell chromatin accessibility sequencing data. Genome Biol 2020;21:94. 32312293 10.1186/s13059-020-02008-0 PMC7169039

[btad505-B42] Zhang Z , Yang C, Zhang X. scDART: integrating unmatched scRNA-seq and scATAC-seq data and learning cross-modality relationship simultaneously. Genome Biol 2022;23:139. 35761403 10.1186/s13059-022-02706-x PMC9238247

[btad505-B43] Zhao J , Wang G, Ming J et al; The Tabula Microcebus Consortium. Adversarial domain translation networks for integrating large-scale atlas-level single-cell datasets. Nat Comput Sci 2022;2:317–30. 10.1038/s43588-022-00251-y 38177826

[btad505-B44] Zheng R , Li M, Liang Z et al SinNLRR: a robust subspace clustering method for cell type detection by non-negative and low-rank representation. Bioinformatics 2019;35:3642–50. 30821315 10.1093/bioinformatics/btz139

[btad505-B45] Zong C , Lu S, Chapman AR et al Genome-wide detection of single-nucleotide and copy-number variations of a single human cell. Science 2012;338:1622–6. 23258894 10.1126/science.1229164 PMC3600412

