# Supplementary material for: scNCL: transferring labels from scRNA-seq to scATAC-seq data with neighborhood contrastive regularization
Source: Bioinformatics. 2023 Aug 16;39(8):btad505. doi: 10.1093/bioinformatics/btad505 (PMC10457667; doi:10.1093/bioinformatics/btad505)
Supplement: btad505_Supplementary_Data [file btad505_supplementary_data.pdf]

SUPPLEMENTARY INFORMATION

scNCL: transferring labels from scRNA-seq to scATAC-seq data with neighborhood contrastive regularization.

Supplementary Figures ------------2-10

Supplementary Notes ------------11-13

Reference ------------14

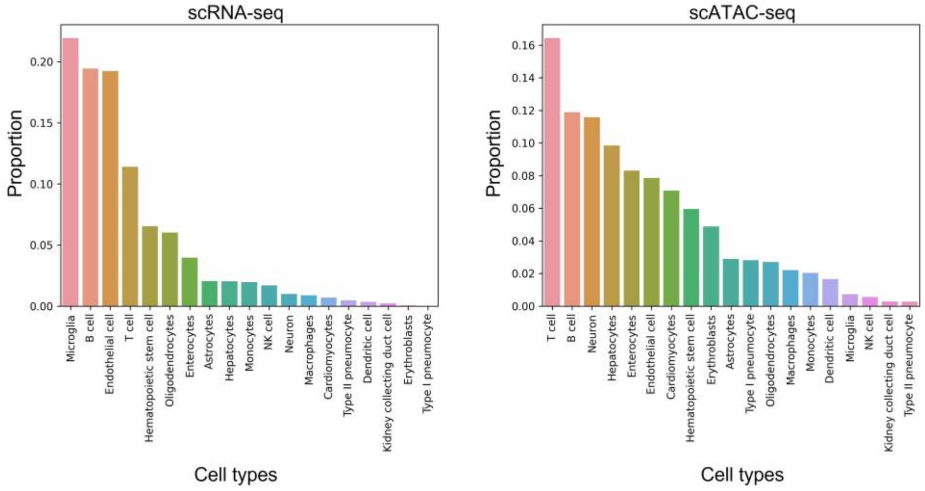

Supplementary Fig. S1. Cell-type composition of scRNA-seq data and scATAC-seq data in MCA-subset dataset.

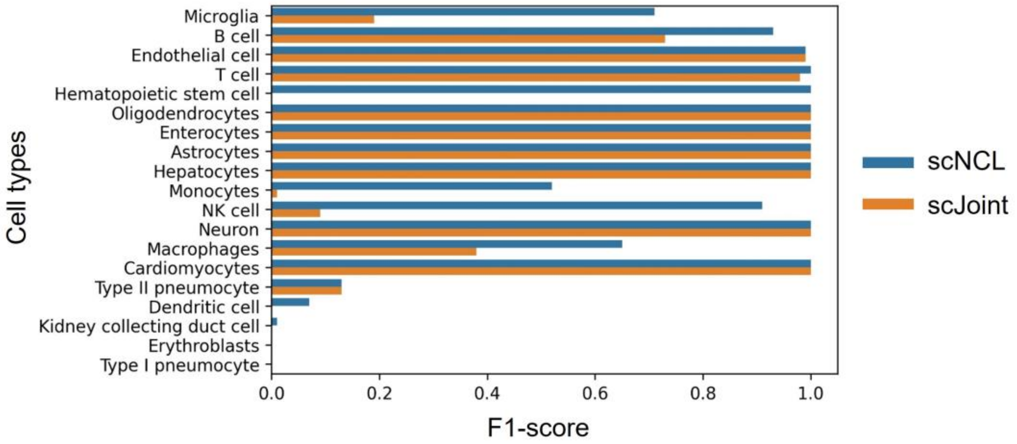

Supplementary Fig. S2. Comparison of cell-type-specific F1 score between scNCL and scJoint for MCA-subset scATAC-seq data. Cell types are sorted bottom-up according to their percentage in the reference (scRNA-seq) data. Microglia accounts for the highest percentage in the reference data.

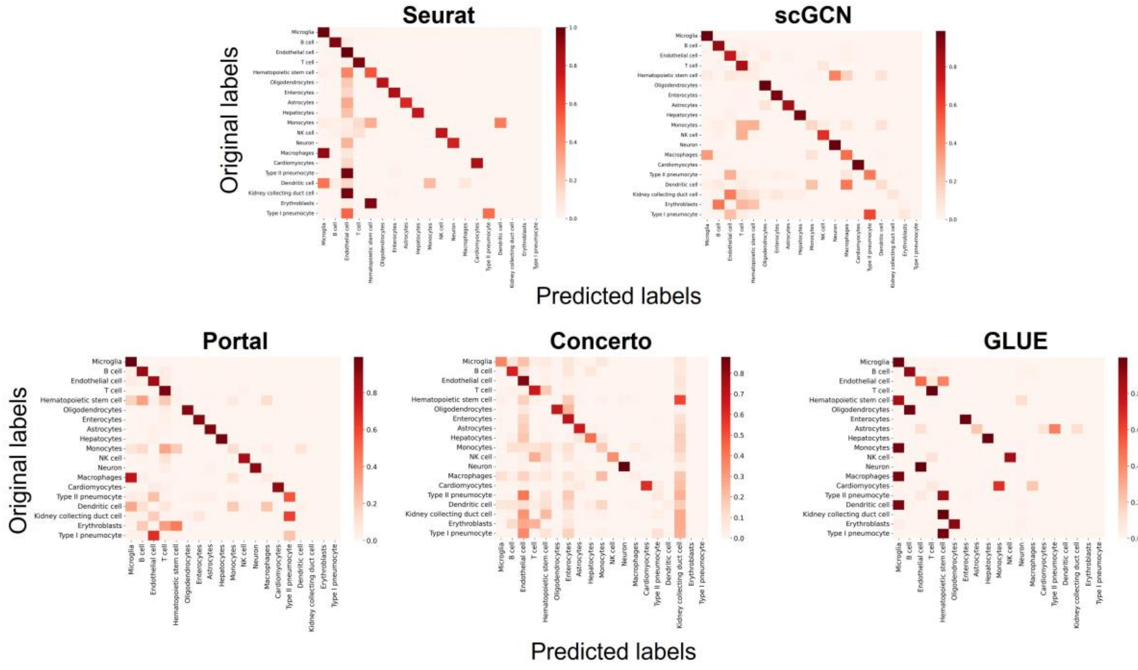

Supplementary Fig. S3. (row normalized) Heatmaps comparing the original labels and transferred labels from Seurat, scGCN, Portal, Concerto, and GLUE for MCA-subset scATAC-seq data. Cell types are sorted bottom-up according to their percentage in the reference (MCA-subset scRNA-seq) data.

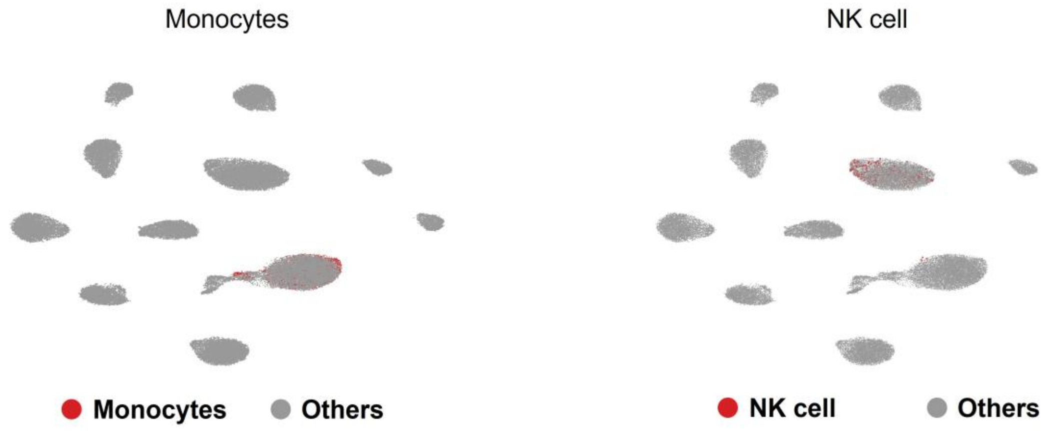

Supplementary Fig. S4. UMAP visualizations of embeddings from scJoint for MCA-subset scATAC-seq data. Monocytes (left) and NK cells (right) both are colored in red. Both cell types are mixed with other major cell types.

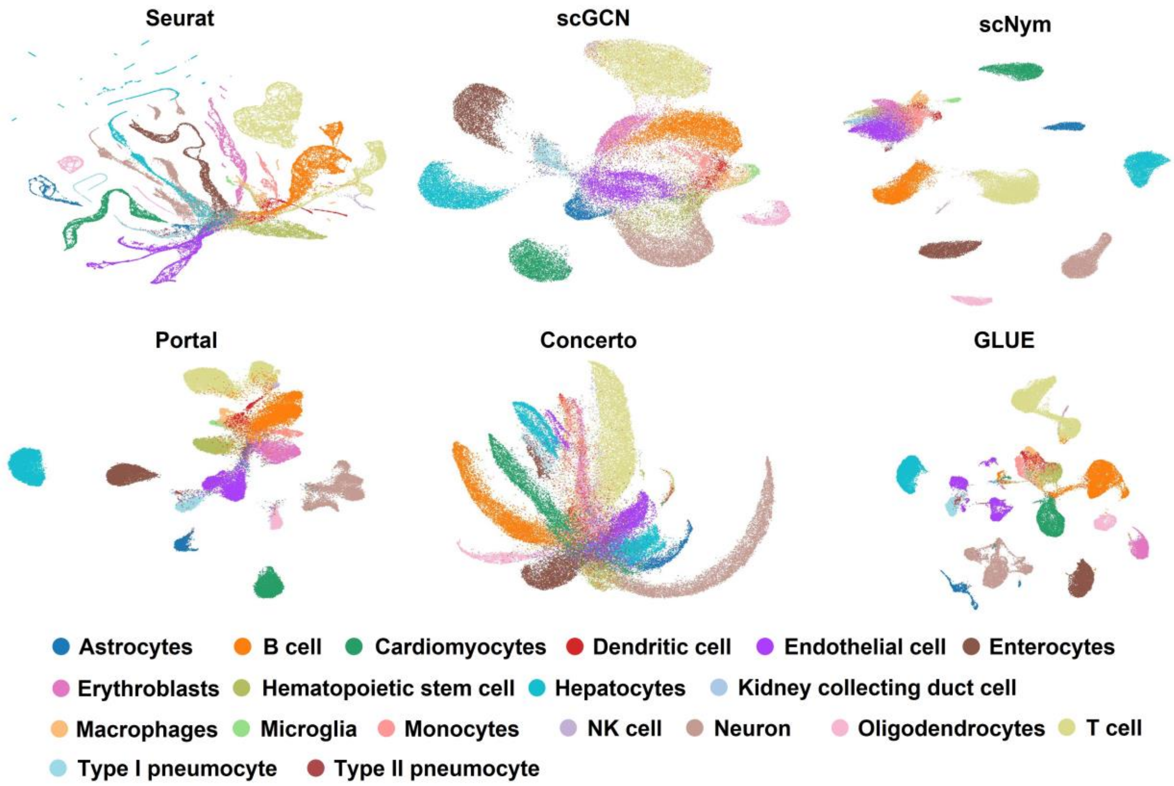

Supplementary Fig. S5. UMAP visualizations of embeddings or imputed data for MCA-subset scATAC-seq data from Seurat, scGCN, scNym, Portal, Concerto, and GLUE. Cells are colored by their cell types.

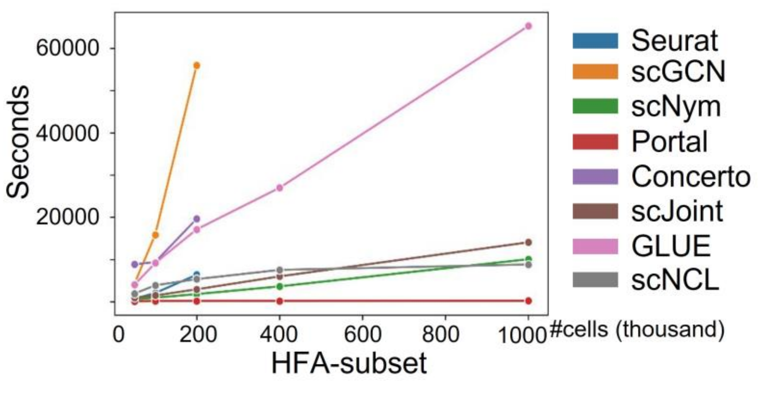

Supplementary Fig. S6. Time consumptions of all methods on HFA-subsets-50k/-100k/-200k/-400k/-full datasets. Note that the time of reading data and preprocessing steps such as LogNormalization, highly variable gene selection, scale, dimension reduction (principal component analysis, PCA and latent semantic indexing, LSI) is not recorded.

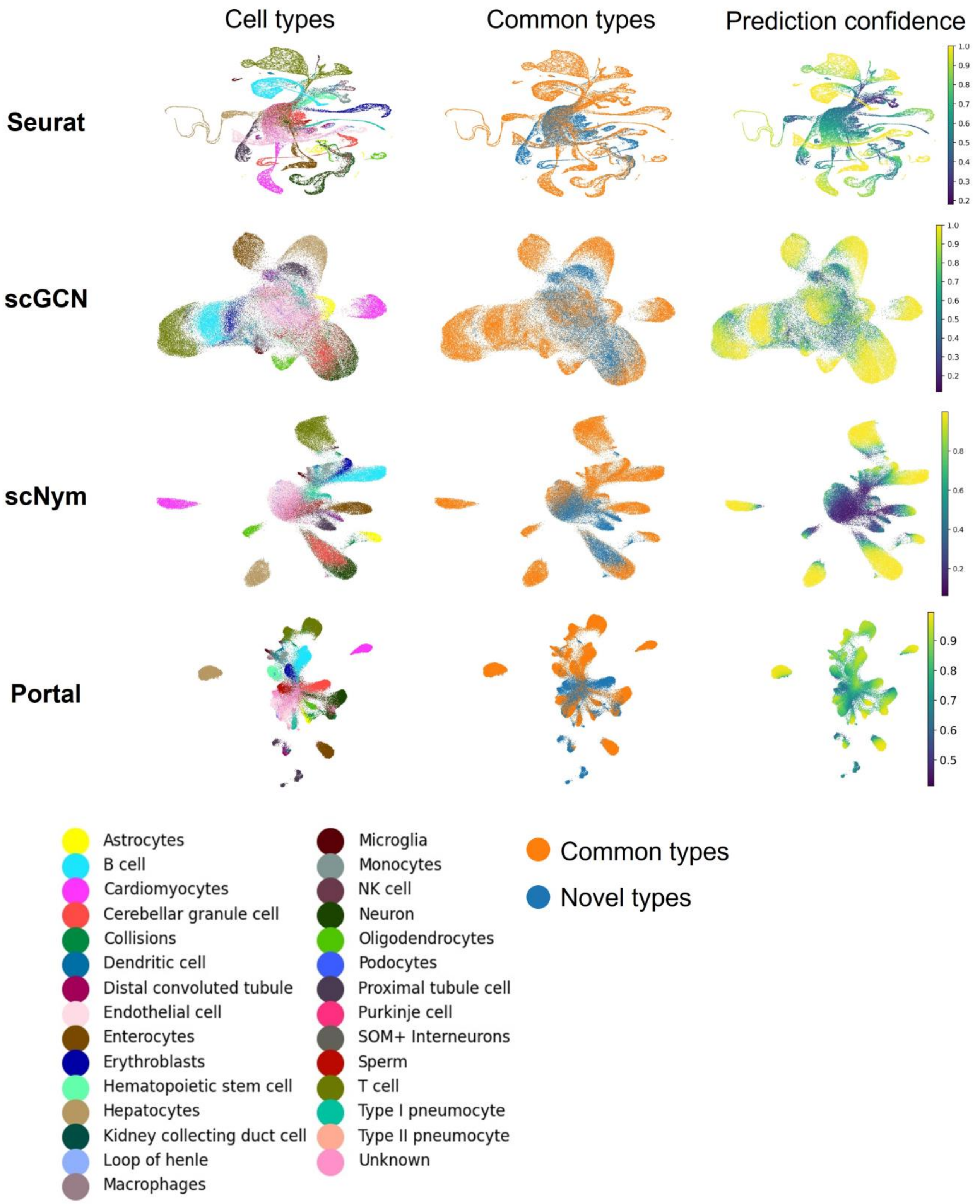

Supplementary Fig. S7a. UMAP visualizations of embeddings or imputed data from Seurat, scGCN, scNym, and Portal for MCAOS scATAC-seq data. Cells are colored by their cell types, common mask, and prediction confidence.

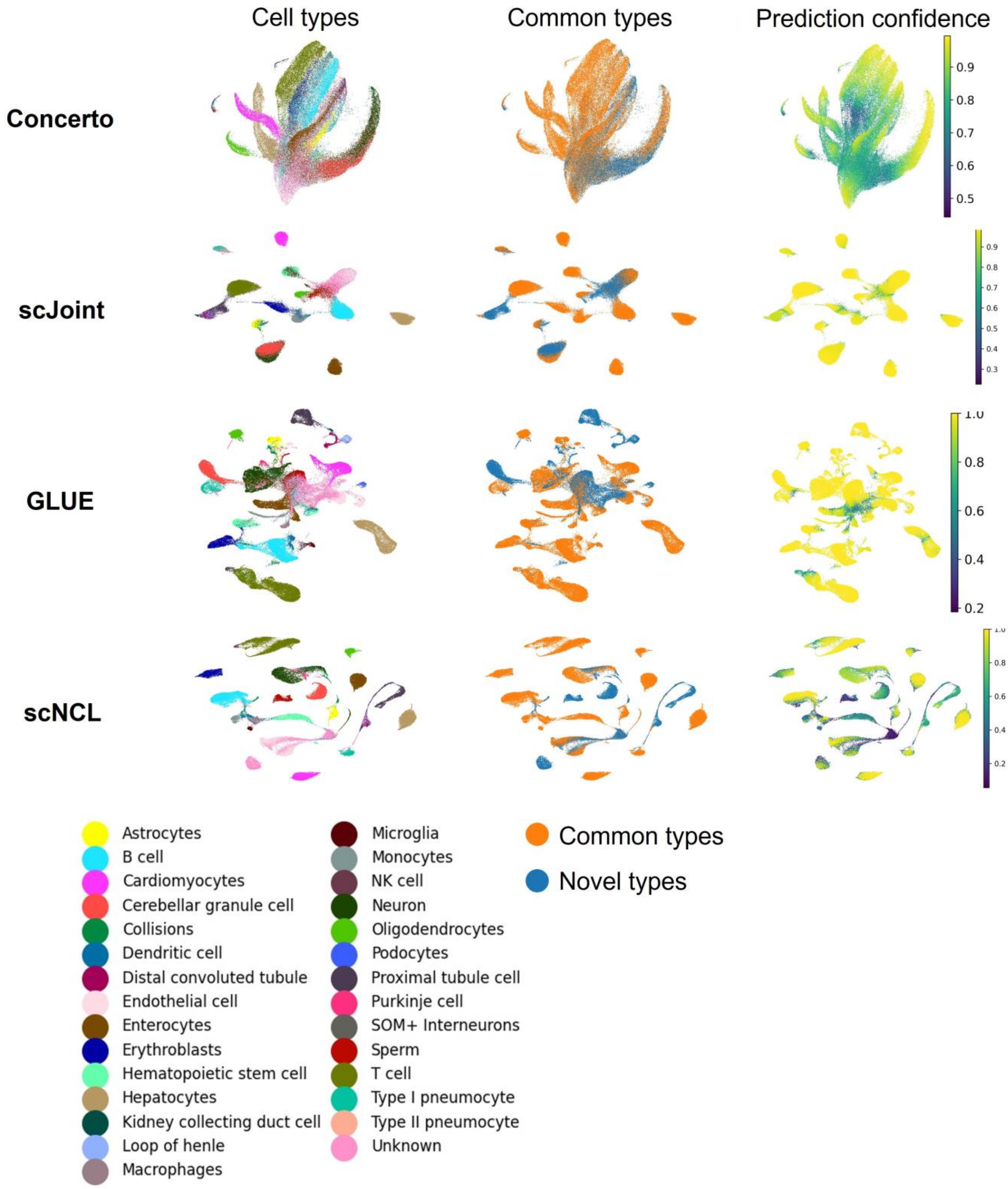

Supplementary Fig. S7b. UMAP visualizations of embeddings or imputed data from Concerto, scJoint, GLUE, and scNCL for MCAOS scATAC-seq data. Cells are colored by their cell types, common mask, and prediction confidence.

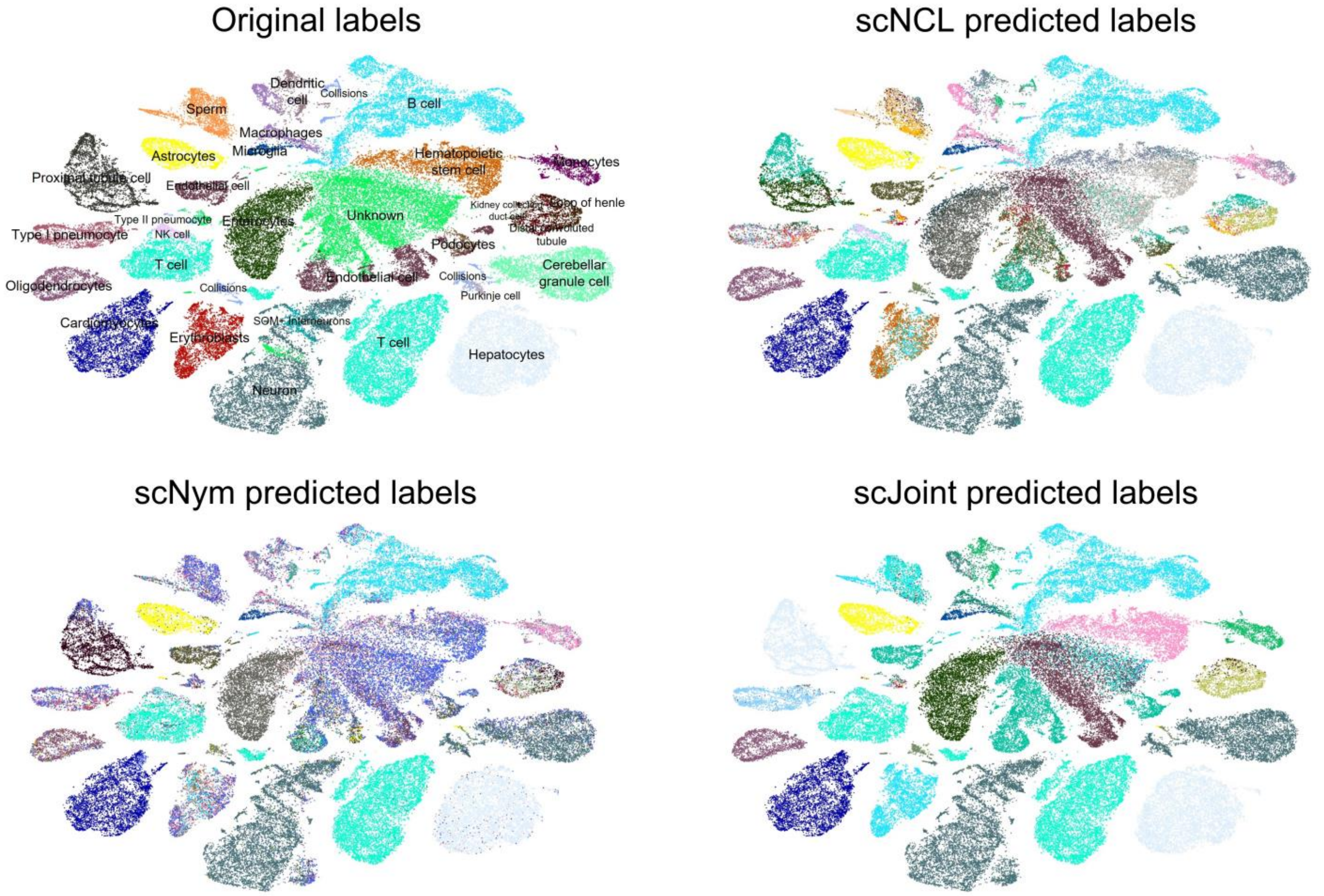

Supplementary Fig. S8. tSNE visualizations of scATAC-seq data in MCA dataset. Cells are colored by original labels (top-left) and predicted labels of scNCL (top-right), scNym (bottom-left), scJoint (bottom-right).

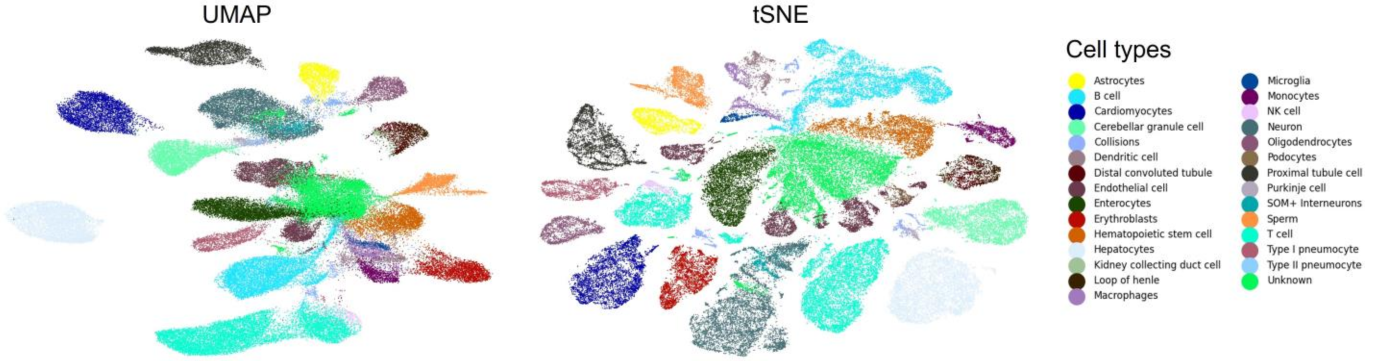

Supplementary Fig. S9. Visualization of cells in MCA scATAC-seq data using UMAP and tSNE as dimensionality reduction tools. Cells are colored by their original annotations.

a

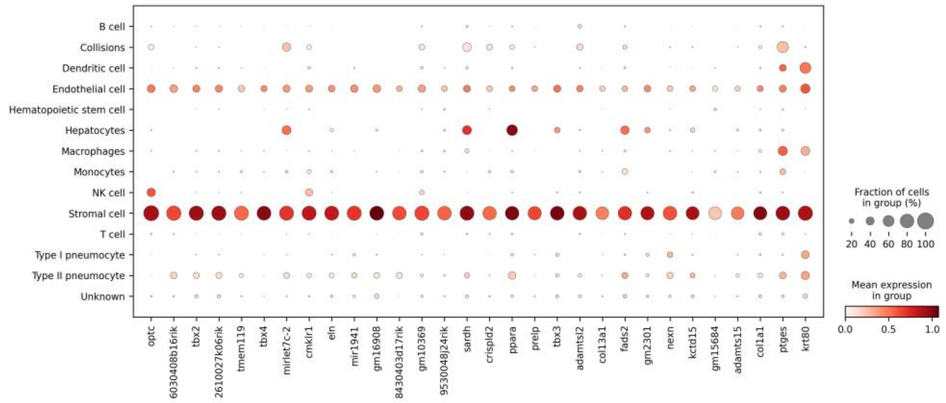

b

	Name	P-value
Go term	Skeletal System Development (GO:0001501)	3.236e-9
	Extracellular Matrix Organization (GO:0030198)	0.000005
	Endochondral Ossification (GO:0001958)	0.00016
	Negative Regulation Of Cytokine Production Involved In Inflammatory Response (GO:1900016)	0.02959
Tabula Muris	Stromal cell in Lung	2.40e-7
	Stromal cell in Trachea	0.008

Supplementary Fig. S10. Upregulated genes in scNCL refined 'stromal cells' and their enrichment analysis. (**a**) Upregulated genes in 'stromal cells'. (**b**) The functional terms enriched in upregulated genes of 'stromal cells' from biological process and their enriched cell types.

a

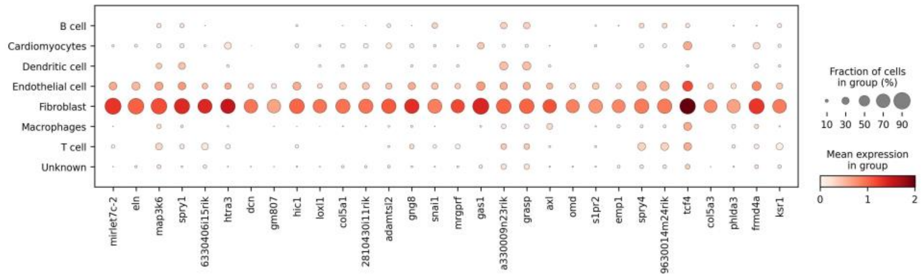

b

	Name	P-value
Go term	Regulation Of Fibroblast Growth Factor Receptor Signaling Pathway (GO:0040036)	0.01884
	Regulation Of Pinocytosis (GO:0048548)	0.00797
	Positive Regulation Of Membrane Depolarization (GO:1904181)	0.00598
	Regulation Of Vitamin D Biosynthetic Process (GO:0060556)	0.00499
Tabula Muris	Fibroblast in Heart	2.86e-8
	Fibroblast in Kidney	0.00008

Supplementary Fig. S11. Upregulated genes in scNCL refined 'fibroblast' and their enrichment analysis. (**a**) Upregulated genes in 'fibroblast'. (**b**) The functional terms enriched in upregulated genes of 'fibroblast' from biological process and their enriched cell types.

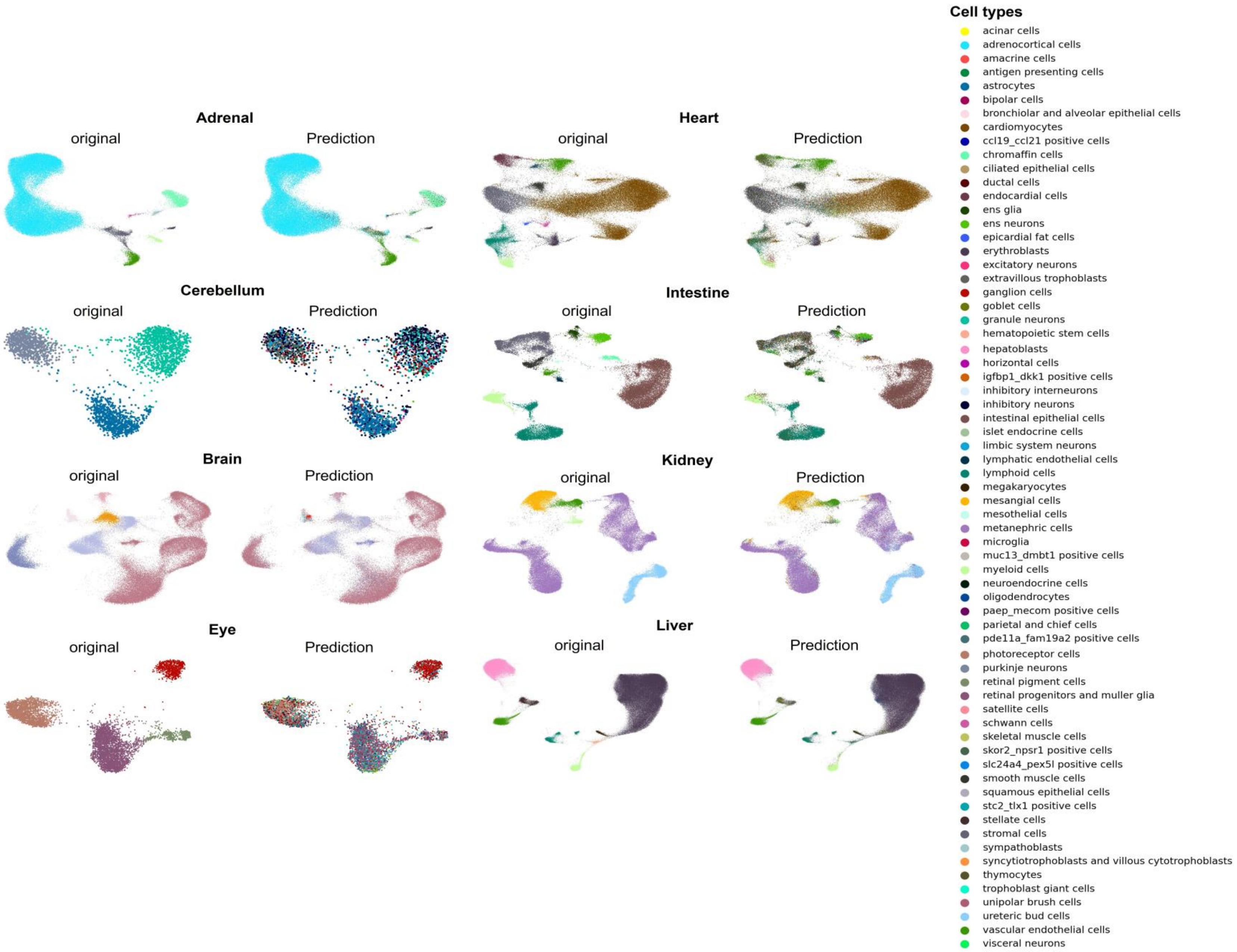

Supplementary Fig. S12a. UMAP visualizations for 15 tissues in scATAC-seq data of HFA dataset. For each tissue, cells are colored by original labels (left) and predicted labels of scNCL (right).

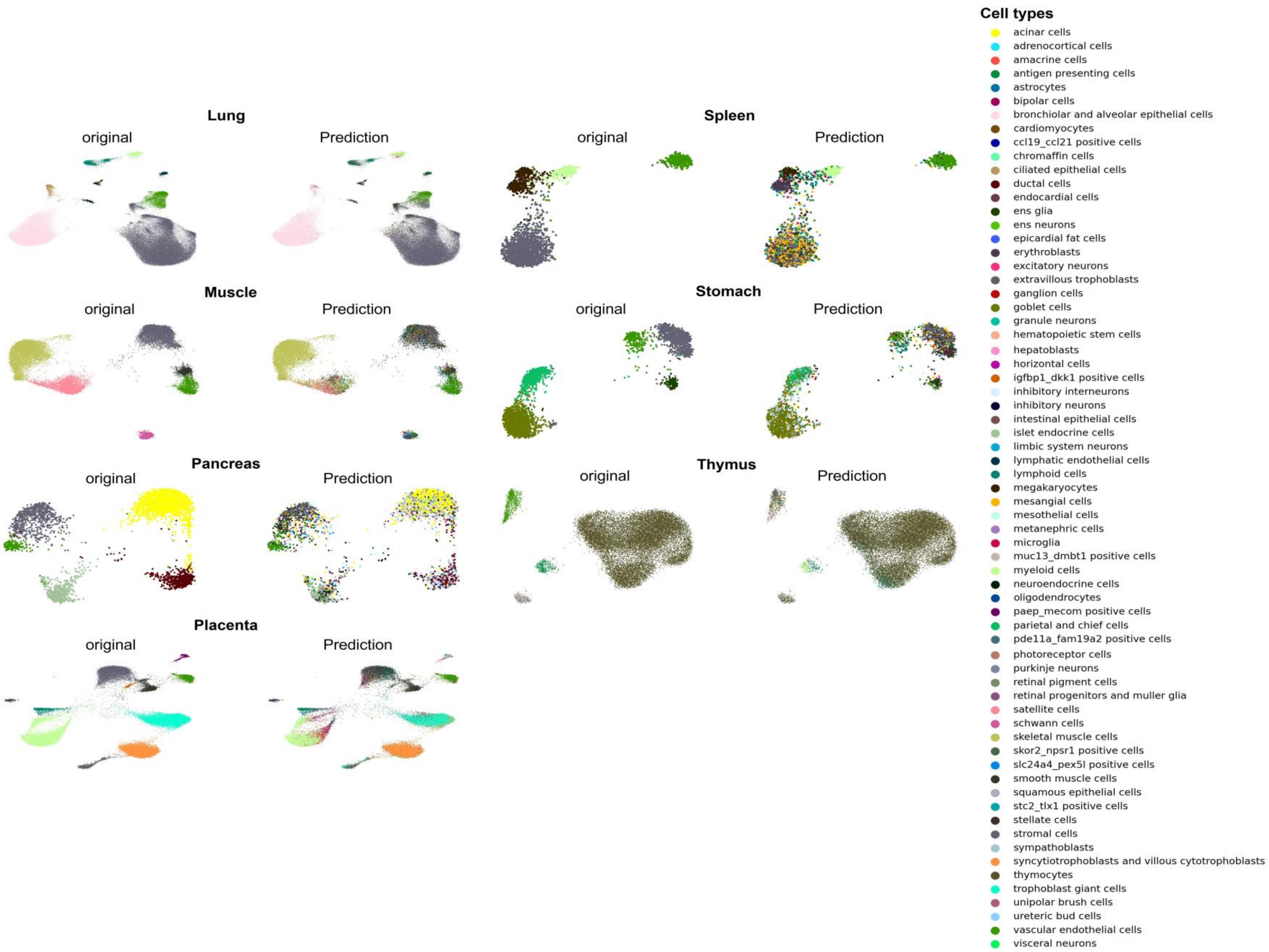

Supplementary Fig. S12b. UMAP visualizations for 15 tissues in scATAC-seq data of HFA dataset. For each tissue, cells are colored by original labels (left) and predicted labels of scNCL (right).

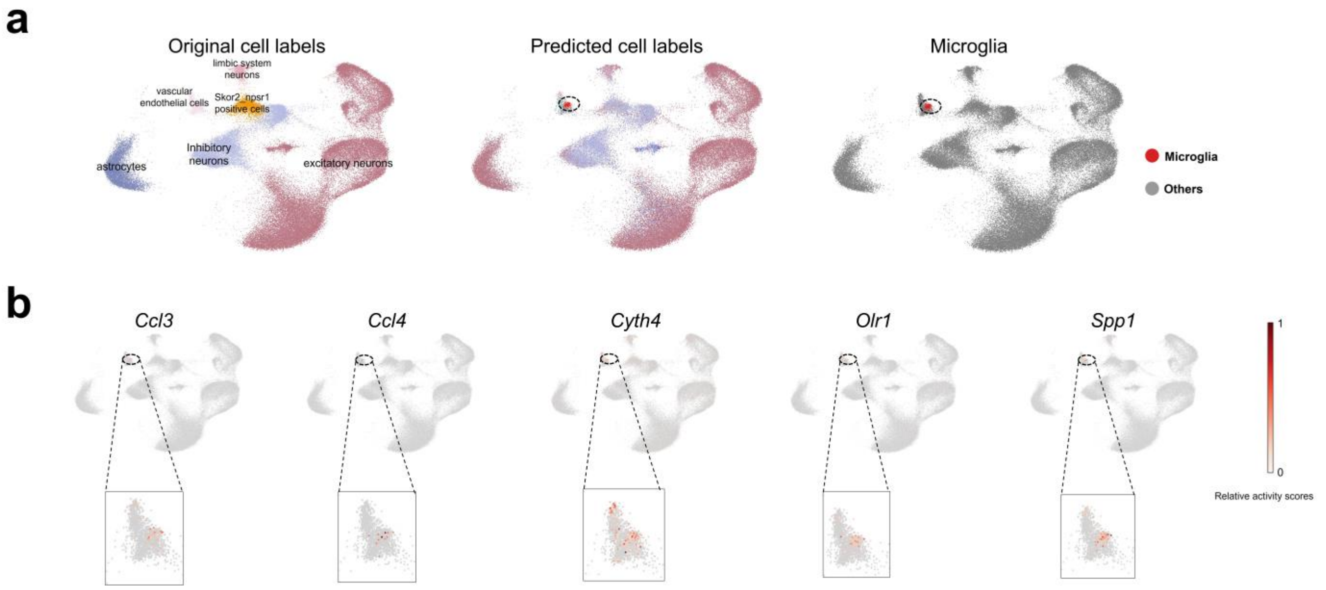

Supplementary Fig. S13. Analysis of scNCL's predictions for cerebrum cells in HFA scATAC-seq data. (a) UMAP visualizations of cerebrum cells. Cells are colored by original labels (left), predicted labels of scNCL (middle), and mask for predicted 'microglia'. (b) Marker expression of 'microglia' in cerebrum, including *Ccl3, Ccl4, Cyth4, Olr1, and Spp1*.

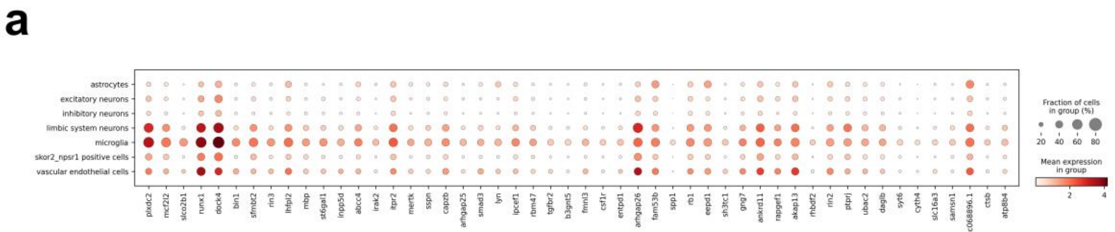

b

	Name	P-value
Go term	Peripheral Nervous System Neuron Development (GO:0048935)	0.01244
	Nerve Growth Factor Signaling Pathway (GO:0038180)	0.02228
	Contact Inhibition (GO:0060242)	0.00001
	Regulation Of Aspartic-Type Endopeptidase Activity Involved In Amyloid Precursor Protein Catabolic Process (GO:1902959)	0.00016
Descartes Cell Types and Tissue 2021	Microglia in Cerebrum	6.16e-16
	Microglia in Cerebellum	1.33e-23

Supplementary Fig. S14. Upregulated genes in scNCL refined 'microglia' and their enrichment analysis. (**a**) Upregulated genes in 'microglia'. (**b**) The functional terms enriched in upregulated genes of 'microglia' from biological process and their enriched cell types.

Supplementary Note

A: Details of methods settings

Seurat: R package Seurat v.4.1.0 [1] was used for all the datasets. The gene expression matrix (GEM) of scRNA-seq and gene activity matrix (GAM) of scATAC-seq were used as input, which were then normalized using the NormalizeData function. Noted that for the CITE-ASAP dataset, the log-normalized GEM/GAM matrix and log-normalized ADTs matrix were concatenated. The FindVariableFeatures function with vst as method was used to select the top 2000 most variable genes from scRNA-seq data. The FindTransferAnchors function was used with 'cca' reduction to identify the anchors between scRNA-seq and scATAC-seq data. Finally, the TransferData function was used to transfer annotations from scRNA-seq to scATAC-seq. The prediction confidence p can be directly exported from the transfer results. The confidence that a cell was predicted as a novel type was calculated using $1-p$.

scGCN: Python package scGCN [2] was used for all the datasets. Following the tutorials, Seurat v4.1.0 was used to prepare the input for scGCN. Specifically, save_processed_data function in data_preprocess_utility.R was used to preprocess raw GEM and GAM, and to construct intra/inter-modality graph. However, we found that scGCN's strategy for selecting highly variable genes was time-consuming. To speed it up, we subsampled the scRNA-seq data to 10000 cells (if its number of cells larger than 10000) during gene selection. Noted that for the CITE-ASAP dataset, the GEM/GAM matrix and ADTs matrix were concatenated before preprocessing. The training and inference processes followed the default pipeline in their repository. For novel type detection, the metrics function in data_preprocess_utility.R was used to compute the entropy score (H) and enrichment score (E) for all scATAC-seq cells. The prediction confidence p was calculated using $E-H$, and the confidence that a cell was predicted as a novel type was calculated using $H-E$.

scNym: Python package scNym v 0.3.2 [3] was used for all the datasets. The GEM of scRNA-seq and GAM of scATAC-seq were preprocessed by normalization (size_factor=1e6) and log transformation, which were then input for training. Note that for the CITE-ASAP dataset, the preprocessed GEM/GAM matrix and log-normalized ADTs matrix were concatenated before training. The scnym.api.scnym_api function is used for training. When scATAC-seq data contains novel cell types that are not existed in scRNA-seq, we set the config parameter to 'new_identity_discovery', otherwise config is set to 'no_new_identity'. The scnym.api.scnym_api function was used for inference. The prediction confidence p was obtained from scNym outputs (saved in 'scNym_confidence' column). The confidence that a cell was predicted as a novel type was calculated using $1-p$.

Concerto: Python package Concerto-reproducibility [4] was used for all the datasets. The GEM of scRNA-seq and GAM of scATAC-seq were used as input. The GEM and GAM were preprocessed using preprocessing_rna function with is_hvg as False. Noted that for the CITE-ASAP dataset, the preprocessed GEM/GAM matrix and ADTs matrix were concatenated before training. Concerto has two usages for label transfer task: query-to-reference mapping and semi-supervised learning. We tried both usages and found that semi-supervised learning generally leads to better transfer performance. Briefly, the preprocessed GEM and GAM were saved into tfrecord format using concerto_make_tfrecord_supervised function. The concerto_train_inter_supervised_uda function was adapted for training and the concerto_test_inter_supervised function was adapted for inference, which produced the integrated latent representations of scRNA-seq and scATAC-seq. Finally, the integrated latent representations were used to annotate scATAC-seq data using knn_classifier function with neighborhood size k as 30. The knn_classifier function not only outputs the predicted labels but also the prediction confidence p. The confidence that a cell was predicted as a novel type was calculated using $1-p$.

Portal: Python package Portal v 1.0.2 [5] was used for all the datasets. The GEM of scRNA-seq and GAM of scATAC-seq were used as input, which then were preprocessed using portal.model.Model.preprocess function. Noted that for the CITE-ASAP dataset, the preprocessed GEM/GAM matrix and log-normalized ADTs matrix were concatenated before training. Then, model was trained and evaluated. Based on the integrated latent representations of scRNA-seq and scATAC-seq, knn_classifier function from Concerto's implementation was used to label scATAC-seq data, which not only outputs the predicted labels but also the prediction confidence p. The confidence that a cell was predicted as a novel type was calculated using $1-p$.

scJoint: Python package scJoint [6] was used for all the datasets. The GEM of scRNA-seq and GAM of scATAC-seq were binarized by zero thresholding. Noted that for the CITE-ASAP dataset, the binarized GEM/GAM matrix and log-transformed normalized ADTs matrix were concatenated before training. The training parameters are referred to the configure notes released in their Github repository. The prediction confidence p was calculated as the description in

original paper. The confidence that a cell was predicted as a novel type was calculated using $1-p$.

GLUE: Python Package GLUE v 0.3.2 [7] was used for all datasets. The GEM of scRNA-seq and chromatin accessibility data of scATAC-seq was used as input. The GEM was preprocessed by normalization, log transformation, scale, and principal component analysis (PCA). The scATAC-seq data was reduced to 100 dimensions using scglue.data.lsi function. To construct a guidance graph of feature interaction, the scglue.data.get_gene_anntation function was used to supplement the coordinate information of genes from GTF files. Then, scglue.genomics.rna_anchored_guidance_graph function was used to compute the guidance graph. The scglue.model.configure_dataset function was used to configure the training and test dataset with default parameters. Noted that training configuration includes the use_cell_type option to supervise classification on scRNA-seq data. However, we observed that using cell type for training led to performance degradation, so we didn't use it. Before training, a subgraph was extracted from the guidance graph. Then, the scglue.models.fit_SCGLUE function was used for training with the extracted subgraph. Finally, the scglue.data.transfer_labels were used to label scATAC-seq data based on the embedded features of scRNA-seq and scATAC-seq data, which also output the prediction confidence p. The confidence that a cell was predicted as a novel type was calculated using $1-p$. For atlas-scale datasets, such as HFA, organ-balanced data preprocessing was performed to balance cell type compositions between atlases. Also, the use_dsc_weight parameter in training configurations was set to 'organ_balancing'.

scNCL: The GEM of scRNA-seq and GAM matrix of scATAC-seq data were binarized by zero thresholding. Noted that for the CITE-ASAP dataset, the binarized GEM/GAM matrix and log-transformed normalized ADTs matrix were concatenated before training. To obtain the low-dimensional representations of raw scATAC-seq data, we adopted different approaches. For the PBMC dataset, following Seurat's tutorial, we computed the dimension-reduced representations (LSI) of raw scATAC-seq data by computing TF-IDF matrix on raw scATAC-seq data and performing SVD decomposition on the TF-IDF matrix. For CITE-ASAP dataset, the ADTs matrix was used as the low-dimensional representations of ASAP data. For MCA dataset, we used tSNE coordinates, which were computed in original study by performing LSI on the peak-by-cell matrix and then performing tSNE, as the low-dimensional representations of raw scATAC-seq data. For HFA dataset and its subsets, we used the top 2-50 PCs, which were computed in original study by performing LSI on the peak-by-cell matrix on each tissue. Noted that on HFA dataset and its subsets, the neighborhood graph for scATAC-seq was calculated separately for each tissue and then aggregated.

B: Ablation study and parameter sensitivity

B1: Ablation study

To compare our proposed projection regularization (PR) loss with the original NNDR loss, we conducted an ablation study by evaluating two different approaches on PBMC, MCA-subset, and HFA-subset-50k datasets: 1) scNCL+NNDR, which used the original NNDR loss proposed by scJoint. 2) scNCL, which used our proposed PR loss. The preparation of MCA-subset and HFA-subset-50k datasets was described in our manuscript. In brief, MCA-subset dataset contains 19726 cells for scRNA-seq and 57563 cells for scATAC-seq. HFA-subset-50k contains 20000 cells for scRNA-seq and 30000 cells for scATAC-seq. Three datasets all have the same collections of cell types between scRNA-seq and scATAC-seq. The evaluation results are shown in Supplementary Table S1. It can be observed that our PR loss improves label transfer performance with a clear margin.

Also, we conducted an ablation study for our introduced neighborhood contrastive learning (NCL) loss by evaluating two different approaches on PBMC, MCA-subset, and HFA-subset-50k datasets: 1) scNCL, which used the NCL loss. 2) scNCL-NCL, which did not use the NCL loss. The results are shown in Supplementary Table S1. It can be observed that without the NCL loss, transfer accuracy dropped dramatically across three datasets.

Overall, our proposed PR loss and NCL loss both contributed to scNCL's superior performance.

Supplementary Table S1. Ablation study on HFA-subset-50k, PBMC, and MCA-subset datasets.

	HFA-subset-50k	PBMC	MCA-subset
scNCL+NNDR	0.8485	0.8452	0.8383
scNCL	0.8518	0.878	0.893
scNCL-NCL	0.6863	0.7761	0.8016

B2: Parameter sensitivity

We examined the sensitivity of scNCL to different parameters choices on HFA-subset-50k and PBMC datasets. On HFA-50k-subset dataset, we set the default values of λ_2, τ, λ_1, p and the dimensionality of embeddings d as 1.0, 0.4, 0.5, 0.8, 64 respectively. On PBMC dataset ,we set the default values of λ_2, τ, λ_1, p and the dimensionality of embeddings d as 0.06, 0.1, 0.05, 0.8, 64 respectively. The evaluation results are shown in Supplementary Fig. S15. Basically, scNCL is highly robust to different training epochs on both datasets. On HFA-50k-dataset, scNCL is insensitive to λ_1 and p. On PBMC dataset, scNCL is insensitive to p and its performance stabilizes at a high level once the λ_1 reaches a certain threshold (e.g., 0.05). In terms of λ_2, scNCL requires it to be above a certain threshold (PBMC: above 0.06, HF-subset-50k: above 0.4). We observed that τ have a relatively important impact on scNCL's performance, and the best choice of τ varies between datasets. However, we found that τ=0.4 leads to stable and good performance. In addition, scNCL is robust with the change in dimensionality of embeddings except for the case where d=32 in HFA-subset-50k dataset, probably because 32 is too small for such a large dataset. Overall, scNCL is generally robust to the choice of different parameters.

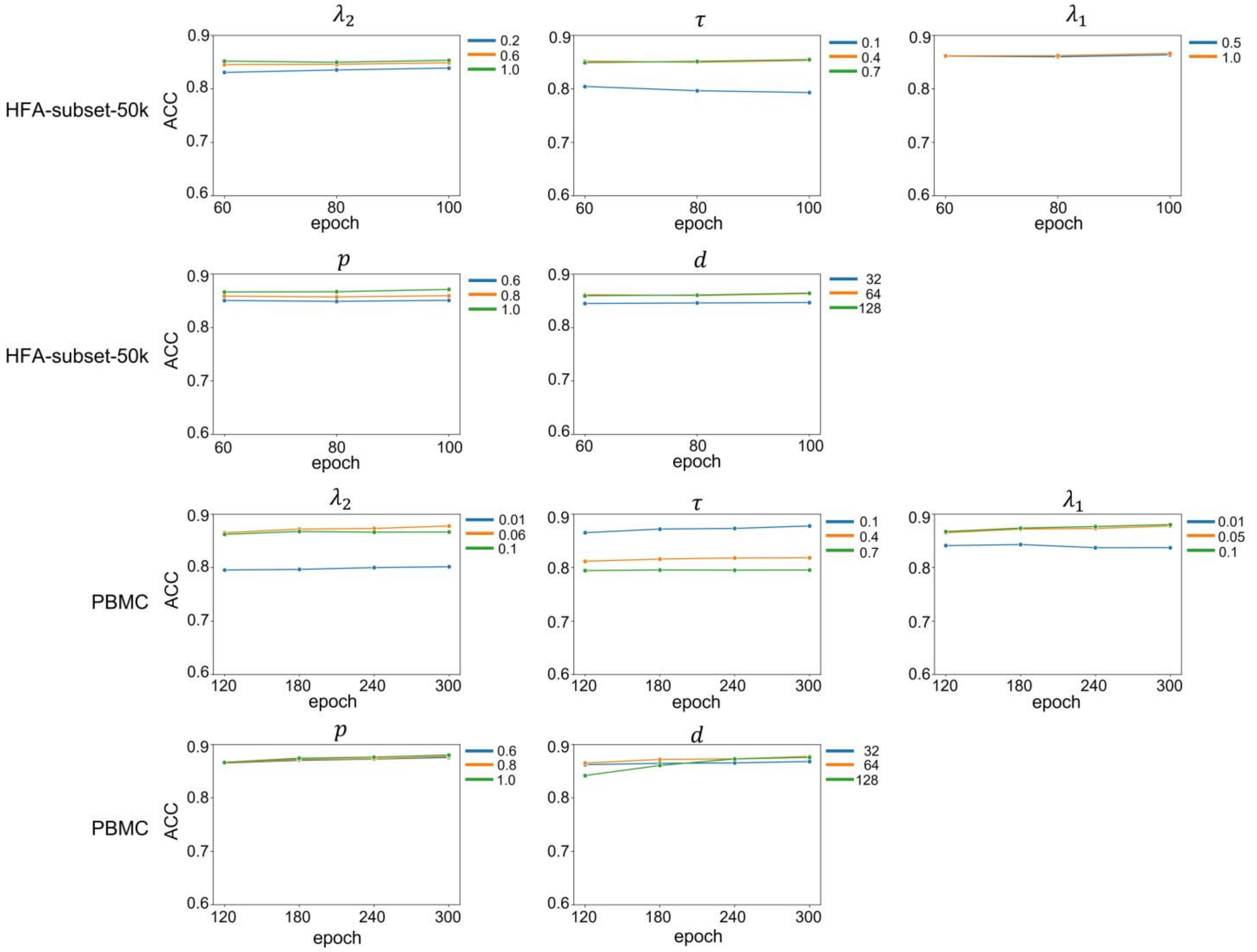

Supplementary Fig. S15. Parameter sensitivity experiments on HFA-subset-50k and PBMC datasets.

Reference

[1] Hao, Yuhan, et al. "Integrated analysis of multimodal single-cell data." *Cell* 184.13 (2021): 3573-3587.

[2] Song, Qianqian, Jing Su, and Wei Zhang. "scGCN is a graph convolutional networks algorithm for knowledge transfer in single cell omics." *Nature communications* 12.1 (2021): 3826.

[3] Kimmel, Jacob C., and David R. Kelley. "Semisupervised adversarial neural networks for single-cell classification." *Genome research* 31.10 (2021): 1781-1793.

[4] Yang, Meng, et al. "Contrastive learning enables rapid mapping to multimodal single-cell atlas of multimillion scale." *Nature Machine Intelligence* 4.8 (2022): 696-709.

[5] Zhao, Jia, et al. "Adversarial domain translation networks for integrating large-scale atlas-level single-cell datasets." *Nature Computational Science* 2.5 (2022): 317-330.

[6] Lin, Yingxin, et al. "scJoint integrates atlas-scale single-cell RNA-seq and ATAC-seq data with transfer learning." *Nature biotechnology* 40.5 (2022): 703-710.

[7] Cao, Zhi-Jie, and Ge Gao. "Multi-omics single-cell data integration and regulatory inference with graph-linked embedding." *Nature Biotechnology* 40.10 (2022): 1458-1466.
